# Klotho Deficiency Impairs Distal Potassium Homeostasis Through mTORC1‐Dependent Suppression of ROMK

**DOI:** 10.1111/apha.70325

**Published:** 2026-10-04

**Authors:** Wakako Kawarazaki, Nobuhiro Ayuzawa, Mitsuhiro Nishimoto, Daigoro Hirohama, Kohei Ueda, Takeshi Marumo, Toshiro Fujita

**Affiliations:** ^1^ Center for Basic Medical Research International University of Health and Welfare (IUHW) Narita Chiba Japan; ^2^ Department of Pharmacology, School of Medicine IUHW Narita Chiba Japan; ^3^ Department of Molecular Biology, School of Medicine IUHW Narita Chiba Japan; ^4^ Division of Clinical Epigenetics, Research Center for Advanced Science and Technology The University of Tokyo Meguro Tokyo Japan; ^5^ Department of Physiology, School of Medicine IUHW Narita Chiba Japan

**Keywords:** aldosterone, distal nephron, hyperkalemia, Klotho, mTORC1, renin–angiotensin system, ROMK

## Abstract

**Aim:**

Hyperkalemia is prevalent in the elderly, and underlying molecular mechanisms remain incompletely understood. We hypothesized that Klotho deficiency, a feature of aging, impairs renal potassium homeostasis and contributes to hyperkalemia.

**Methods:**

Klotho heterozygous knockout (KLhet) mice, characterized by reduced Klotho levels, were used to investigate determinants of potassium handling, including renal outer medullary potassium channel (ROMK)1, the epithelial sodium channel (ENaC), which provides the driving force for ROMK‐mediated potassium secretion, and plasma aldosterone concentration (PAC).

**Results:**

KLhet mice developed hyperkalemia with reduced urinary potassium excretion, accompanied by activation of renal mechanistic target of rapamycin complex 1 (mTORC1) signaling, indicated by increased phosphorylated S6, and decreased ROMK1 abundance in the plasma membrane fraction. Adrenal expression of aldosterone synthase *Cyp11b2* and PAC were elevated, and ENaC activity was enhanced, as evidenced by increased cleavage of α‐ and γ‐ENaC subunits, partially compensating for impaired potassium excretion. Pharmacological ENaC inhibition with amiloride precipitated severe hyperkalemia in KLhet mice. In contrast, treatment with the mTORC1 inhibitor rapamycin or Klotho supplementation suppressed renal mTORC1 signaling, restored plasma membrane ROMK1 abundance, increased urinary potassium excretion, and normalized serum potassium levels, with reduced *Cyp11b2* expression.

**Conclusion:**

Klotho deficiency impairs renal potassium handling through mTORC1‐dependent downregulation of ROMK, with compensatory hyperaldosteronism and ENaC activation. These findings highlight an important role of the Klotho–mTORC1 axis in distal potassium homeostasis and its potential as a therapeutic target for Klotho deficiency–associated hyperkalemia.

## Practioner Points

- Klotho deficiency impaired urinary potassium excretion and caused hyperkalemia, accompanied by activation of renal mTORC1 signaling and reduced plasma membrane abundance of ROMK in the distal nephron.
- These alterations were associated with compensatory hyperaldosteronism and ENaC activation, while pharmacological ENaC inhibition further exacerbated hyperkalemia.
- Both mTORC1 inhibition with rapamycin and Klotho supplementation restored plasma membrane ROMK abundance in the distal nephron, improved potassium excretion, and normalized serum potassium levels, highlighting an important role of the Klotho–mTORC1–ROMK axis in renal potassium homeostasis.

## 1 Introduction

Hyperkalemia is a serious and potentially life‐threatening electrolyte disorder and a marker of poor prognosis [1]. Older adults are particularly susceptible due to impaired renal potassium excretion associated with aging‐related declines in glomerular filtration rate and tubular secretory capacity [2, 3, 4, 5]. Although the renin–angiotensin–aldosterone system plays a key role in potassium homeostasis, the mechanisms underlying hyperkalemia in aging remain incompletely understood [6].

Klotho, an anti‐aging protein predominantly expressed in the kidney, declines with age and has diverse systemic effects [7, 8, 9, 10]. Klotho heterozygous knockout (KLhet) mice exhibit circulating Klotho levels comparable to aged mice without overt aging phenotypes, making them a suitable model to study the effects of Klotho deficiency independent of aging‐related confounders [11]. Previous studies have shown that soluble Klotho exerts protective effects in the kidney [12, 13] and increases the membrane abundance of renal ion channels, including transient receptor potential cation channel subfamily V member 5 (TRPV5) and the renal outer medullary potassium channel (ROMK)1, in vitro [14, 15]. However, whether Klotho deficiency alters renal potassium handling in vivo remains unclear.

Renal potassium secretion is primarily mediated by ROMK and large‐conductance calcium‐activated potassium (BK) channels [16, 17]. ROMK plays a central role in basal potassium secretion, particularly in the aldosterone‐sensitive distal nephron, where it is functionally coupled with epithelial sodium channels (ENaC). Disruption of this ENaC–ROMK axis may impair potassium excretion.

Importantly, the physiological role of ROMK differs along the nephron. In the thick ascending limb (TAL), ROMK mediates potassium recycling required for NaCl reabsorption, whereas in the distal nephron, particularly in the distal convoluted tubule Segment 2 (DCT2), connecting tubule (CNT), and cortical collecting duct (CCD), it serves as the principal pathway for regulated urinary potassium secretion [16]. Among ROMK isoforms, ROMK1 is enriched in the aldosterone‐sensitive distal nephron and is most directly involved in potassium secretion, whereas ROMK2 and ROMK3 are also expressed in the TAL [16]. We therefore focused on ROMK1 to assess distal potassium handling.

The mechanistic target of rapamycin (mTOR) is a key regulator of cellular metabolism and has been implicated in renal electrolyte transport. Activation of mTORC1 suppresses ROMK1 activity [18], whereas mTORC2 enhances ENaC activity through SGK1‐dependent mechanisms [19]. In addition, hyperkalemia itself has been reported to activate mTORC2 signaling, thereby enhancing ENaC activity and distal sodium reabsorption [19]. Thus, both mTORC1 and mTORC2 pathways may contribute to altered potassium handling in the setting of Klotho deficiency and hyperkalemia. Klotho deficiency has been shown to activate mTORC1 signaling [20, 21, 22]. However, whether mTORC1 signaling contributes to potassium dysregulation in Klotho‐deficient states remains unknown.

In the present study, we aimed to determine whether Klotho deficiency impairs renal potassium handling and to examine the role of mTORC1 signaling in this process using KLhet mice.

## 2 Methods

### 2.1 Approval for Animal Experiments

All methods were carried out in accordance with the ARRIVE guidelines. Animal care and treatment complied with the standards described in the Guidelines for the Care and Use of Laboratory Animals of the University of Tokyo and the International University of Health and Welfare (IUHW). All experiments were approved by the Institutional Animal Care and Use Committee (approval numbers RAC190009‐2 in the University of Tokyo, and 22022NA and 26004NA in IUHW).

### 2.2 Animals

Male C57BL/6JJcl wild‐type (WT) and Klotho heterozygous knockout (KLhet; α‐Klotho KO/Jcl) mice (CLEA Japan, Tokyo, Japan) were housed under standard conditions (12‐h light/dark cycle, 22°C). Sixteen‐week‐old mice were used. Age‐matched C57BL/6JJcl mice were used as WT controls because they share the same genetic background strain as α‐Klotho KO/Jcl mice. WT and KLhet mice were bred separately and were not littermates.

Four experimental protocols were performed: (1) WT and KLhet mice (*n* = 13 each); (2) WT (*n* = 8), KLhet (*n* = 9), and KLhet mice treated with rapamycin (1 mg/kg/day for 2 weeks; *n* = 8); (3) WT, vehicle‐treated KLhet, and Klotho‐supplemented KLhet mice (*n* = 7 each); and (4) WT (*n* = 7), KLhet (*n* = 7), WT mice treated with amiloride (*n* = 6), and KLhet mice treated with amiloride (*n* = 6), with amiloride administered at 0.35 mg/kg/day in drinking water. In an additional control experiment, WT mice were treated with rapamycin using the same protocol (WT, *n* = 7; WT + rapamycin, *n* = 7) to assess the effects of rapamycin on renal potassium handling in WT mice. Klotho supplementation was achieved by in vivo transfection of a plasmid encoding full‐length mouse Klotho as previously described [11]. All mice were fed a standard laboratory chow (MF diet; Oriental Yeast Co. Ltd., Tokyo, Japan) containing 0.21% sodium and 0.99% potassium (0.21 g Na and 0.99 g K per 100 g diet), and had free access to food and water. Twenty‐four‐hour urine was collected using metabolic cages. Blood samples were obtained under isoflurane anesthesia, and electrolytes were measured using an i‐STAT1 analyzer (Abbott) [23, 24]. After sacrifice, kidneys and adrenal glands were harvested. Plasma aldosterone levels were measured by ELISA (Enzo Life Sciences Inc., Farmingdale, NY, USA; ADI‐900‐173). Fractional excretion of sodium (FENa) and potassium (FEK) were calculated as previously described [25]. The transtubular potassium gradient (TTKG) was calculated as follows: TTKG = (urine potassium/plasma potassium)/(urine osmolality/plasma osmolality). Primary outcome measures included serum potassium levels and urinary potassium excretion. Klotho expression was assessed at the mRNA and protein levels by real‐time PCR and Western blotting. Serum Klotho protein levels were determined by immunoprecipitation followed by Western blotting as previously described [11].

### 2.3 Real‐Time Reverse Transcription–Polymerase Chain Reaction

Total RNA was extracted from whole kidneys and adrenal glands using the RNeasy Mini Kit (Qiagen) [23, 24]. RNA concentration and purity were assessed using a NanoDrop spectrophotometer (Thermo Fisher Scientific). Complementary DNA was synthesized using the High‐Capacity cDNA Reverse Transcription Kit (Thermo Fisher Scientific). Renal *Klotho* and adrenal *Cyp11b1* and *Cyp11b2* mRNA expression was quantified by TaqMan‐based real‐time PCR using a StepOnePlus Real‐Time PCR System (Applied Biosystems).

### 2.4 Western Blot Analysis

Protein expression of ROMK1, α‐, β‐, and γ‐subunits of ENaC, BKα, Na^+^–Cl^−^ cotransporter (NCC), and NCC phosphorylated at threonine 53 (pT53‐NCC) was assessed in plasma membrane fractions isolated from kidneys using a commercially available kit (Minute Plasma Membrane Protein Isolation Kit, SM‐005; Invent Biotechnologies Inc., Plymouth, MN, USA) [26]. Pan‐cadherin was used as a loading control for plasma membrane proteins. The plasma membrane fraction was separated from the organelle membrane, cytosolic, and nuclear fractions according to the manufacturer's protocol, and only the isolated plasma membrane fraction was used for Western blot analysis.

Expression of S6, phosphorylated (p‐) S6, Akt, phosphorylated Akt (p‐Akt), Klotho, and GAPDH was analyzed in whole‐kidney lysates. Western blotting was performed as described previously [11]. Briefly, whole‐kidney lysates and isolated plasma membrane fractions were subjected to SDS–PAGE and immunoblotting. Signals were detected by enhanced chemiluminescence and quantified using an ImageQuant LAS 4000 mini system (Fujifilm, Tokyo, Japan). Details of primary antibodies are provided in Table S1. The specificity of the BKα (KCa1.1) antibody (APC‐107, Alomone Labs) is supported by a previous study using tissues from global KCNMA1‐knockout mice [27].

For immunoblot analysis, plasma membrane fractions were prepared from whole kidney tissue; therefore, segment‐specific differences could not be distinguished. Because ROMK1 is predominantly expressed in the distal nephron, an anti‐ROMK1 antibody was used to assess plasma membrane abundance of ROMK1. In contrast, for immunofluorescence studies, a pan‐ROMK antibody was used in combination with segment‐specific markers to assess the localization of ROMK in distal nephron segments.

### 2.5 Immunofluorescence Staining

Immunofluorescence staining was performed as previously described with minor modifications [24]. Kidneys were fixed overnight in 4% paraformaldehyde at 4°C, cryoprotected in 30% sucrose solution, embedded in OCT compound (Sakura Finetek, Tokyo, Japan), and frozen. Cryosections (6 μm) were prepared, subjected to antigen retrieval in citrate buffer (pH 6.0), and blocked with 5% normal donkey serum. Sections were incubated with primary antibodies followed by Alexa Fluor 488‐ or 555‐conjugated donkey secondary antibodies (Invitrogen). Double immunofluorescence staining was performed sequentially with an additional blocking step between stainings. Details of primary antibodies are provided in Table S1. Images were acquired using an LSM 800 confocal laser scanning microscope (Carl Zeiss AG, Oberkochen, Germany).

### 2.6 Statistical Analysis

Data are presented as the mean ± standard error of the mean (SEM). Comparisons between two independent groups were performed using Welch's *t*‐test. For comparisons among three independent groups, one‐way analysis of variance (ANOVA) followed by Holm–Bonferroni‐adjusted pairwise comparisons was used. A *p*‐value of < 0.05 was considered statistically significant. All analyses were performed using JMP Pro software, version 19.0.3 (SAS Institute Inc., Cary, NC, USA).

## 3 Results

### 3.1 Elevated Serum Potassium and Reduced Urinary Potassium Excretion in KLhet Mice

Compared with WT mice, KLhet mice showed an approximately 50% reduction in renal Klotho mRNA expression (Figure 1A) and markedly reduced protein expression (Figure 1B). Serum Klotho protein was also markedly decreased in KLhet mice (Figure 1C). Serum sodium level was unchanged between groups; however, serum potassium levels were significantly higher in KLhet mice (Figure 1D). Serum potassium increased from 4.0 ± 0.03 mEq/L in WT mice to 4.7 ± 0.2 mEq/L in KLhet mice (*p* < 0.001). Serum creatinine levels and FENa were comparable between groups. Twenty‐four‐hour urine volume was also unchanged (WT, 1.68 ± 0.30 mL vs. KLhet, 1.46 ± 0.17 mL; *p* = 0.52), whereas FEK was significantly decreased in KLhet mice (WT 27.5 ± 2.1 vs. KLhet 12.8% ± 1.3%, *p* < 0.001; Figure 1E). The transtubular potassium gradient (TTKG) was also significantly lower in KLhet mice than in WT mice, further supporting impaired distal potassium secretion (WT 9.9 ± 1.4 vs. KLhet 5.3 ± 0.7, *p* < 0.05; Figure 1E). Accordingly, the urinary Na^+^/K^+^ ratio was increased in KLhet mice (Figure 1E). Serum calcium, phosphate, and 1,25‐dihydroxyvitamin D_3_ [1,25‐(OH)_2_D_3_] levels were comparable between WT and KLhet mice (Figure S1). Thus, no overt alterations in calcium, phosphate, or vitamin D metabolism were observed in KLhet mice under the conditions of the present study.

**FIGURE 1:**
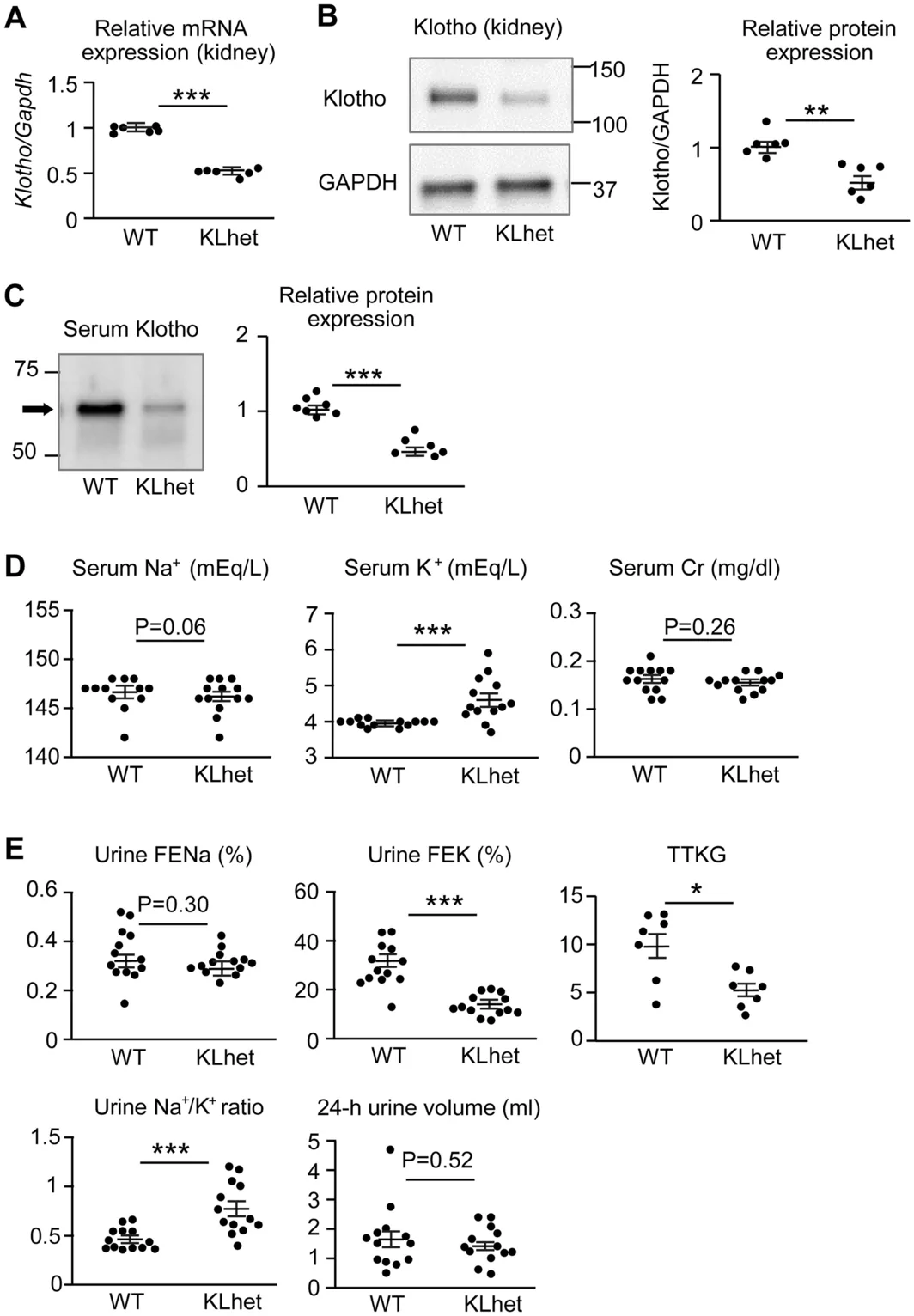
Reduced Klotho expression, hyperkalemia, and impaired urinary potassium excretion in Klotho heterozygous knockout (KLhet) mice. (A) Relative renal *Klotho* mRNA expression in wild‐type (WT) mice and KLhet mice (*n* = 6 per group). *Gapdh* was used as an internal control. (B) Representative Western blot and quantification of renal Klotho protein expression in WT and KLhet mice (*n* = 6 per group). GAPDH was used as a loading control. (C) Representative Western blot and quantification of serum Klotho protein levels in WT mice (*n* = 7) and KLhet mice (*n* = 6). (D) Serum sodium (Na^+^), potassium (K^+^), and creatinine (Cr) concentrations in WT and KLhet mice (*n* = 13 per group). (E) Urinary fractional excretion of sodium (FENa), fractional excretion of potassium (FEK), transtubular potassium gradient (TTKG), urinary sodium‐to‐potassium (Na^+^/K^+^) ratio, and 24‐h urine volume in WT and KLhet mice. For FENa, FEK, urinary Na^+^/K^+^ ratio, and 24‐h urine volume, *n* = 13 per group; for TTKG, *n* = 7 per group. Data are presented as mean ± SEM. *p < 0.05, **p < 0.01, ***p < 0.001 versus WT mice.

### 3.2 Reduced Plasma Membrane ROMK1 Expression With Compensatory ENaC Activation in KLhet Mice

Consistent with reduced urinary potassium excretion, the complex‐glycosylated, core‐glycosylated, and unglycosylated forms of ROMK1 in the isolated plasma membrane fraction were reduced in KLhet mice (Figure 2A). Western blot analysis showed increased cleavage of α‐ and γ‐ENaC in the plasma membrane fraction of KLhet kidneys, indicating enhanced ENaC activation (Figure 2B). Plasma aldosterone concentration (PAC) was significantly elevated in KLhet mice (WT: 288.0 ± 22.9 vs. KLhet: 435.8 ± 33.3 pg/mL, *p* = 0.004; Figure 2C). Consistent with this, administration of the ENaC inhibitor amiloride further increased serum potassium levels in KLhet mice, resulting in marked hyperkalemia (from 4.7 ± 0.2 to 5.8 ± 0.4 mEq/L, *p* = 0.046), whereas no significant change was observed in WT mice (Figure 2D).

**FIGURE 2:**
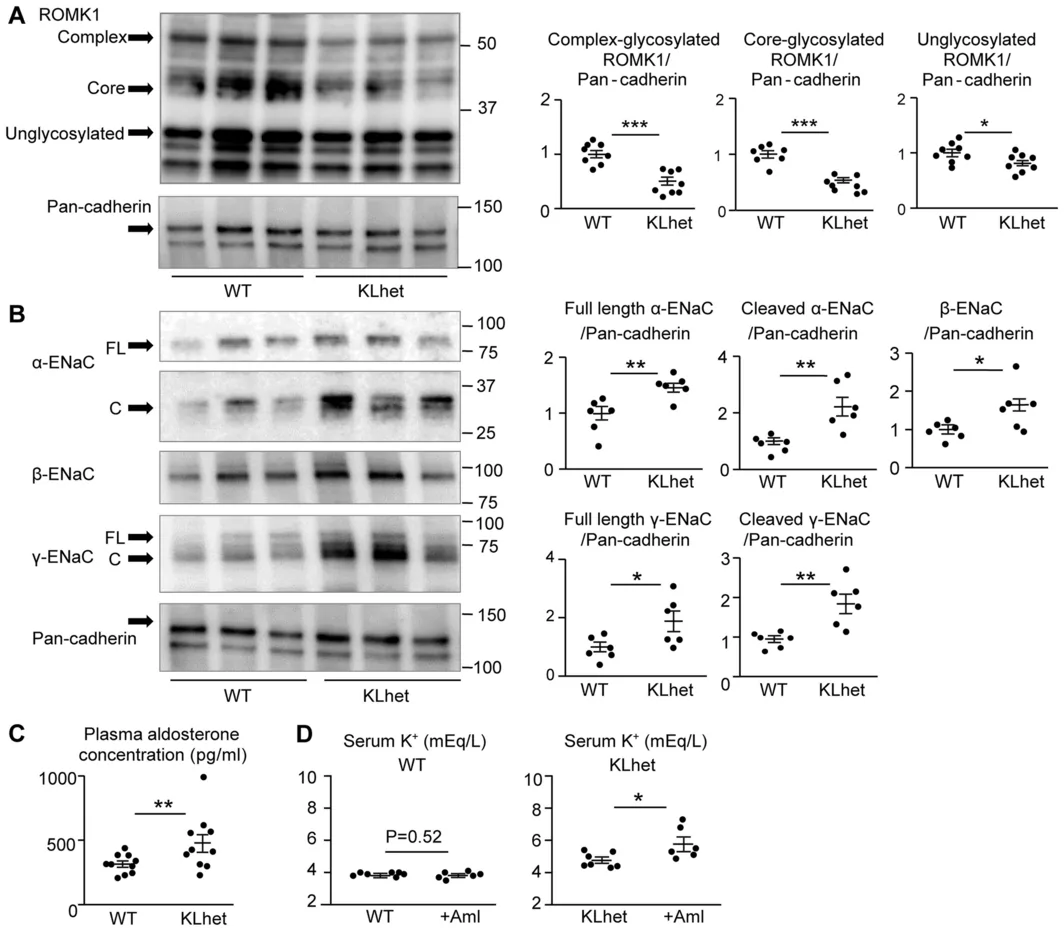
Reduced plasma membrane ROMK1 expression and enhanced ENaC activation in KLhet mice, with exacerbation of hyperkalemia by amiloride. (A) Representative Western blot and quantification of ROMK1 protein expression in renal plasma membrane fractions from WT and KLhet mice. Complex‐glycosylated (approximately 50–55 kDa), core‐glycosylated (approximately 40 kDa), and unglycosylated forms of ROMK1 are shown and quantified (*n* = 8 per group). (B) Representative Western blot and quantification of α‐, β‐, and γ‐subunits of ENaC in renal plasma membrane fractions from WT and KLhet mice. Full‐length (FL) and cleaved (C) forms of α‐ENaC (85–90 kDa; 30–35 kDa) and γ‐ENaC (80–85 kDa; 70–75 kDa), and full‐length β‐ENaC (85–95 kDa) are shown (*n* = 6 per group). (C) Plasma aldosterone concentrations in WT and KLhet mice (*n* = 10 per group). (D) Serum potassium (K^+^) concentrations in WT (*n* = 7), WT + Aml (*n* = 6), KLhet (*n* = 7), and KLhet + Aml (*n* = 6). Data are presented as mean ± SEM. Welch's *t*‐test was used for comparisons between two groups. *p < 0.05, **p < 0.01, ***p < 0.001, as indicated. Aml, amiloride; C, cleaved; FL, full‐length.

### 3.3 mTORC1 Inhibition by Rapamycin Restores ROMK1 Abundance and Corrects Hyperkalemia in KLhet Mice

mTOR signaling has been implicated in tubular potassium handling [28], and previous studies have suggested that Klotho can suppress mTOR signaling [29]. Compared with WT mice, KLhet mice exhibited significantly increased phosphorylated S6 (p‐S6), a downstream target of mTORC1, whereas phosphorylated Akt (Ser473), a commonly used readout of mTORC2 activity, remained unchanged (Figure 3A,B), supporting predominant activation of mTORC1 without detectable activation of mTORC2. Treatment with the mTORC1 inhibitor rapamycin normalized p‐S6 levels in KLhet mice (Figure 3C).

**FIGURE 3:**
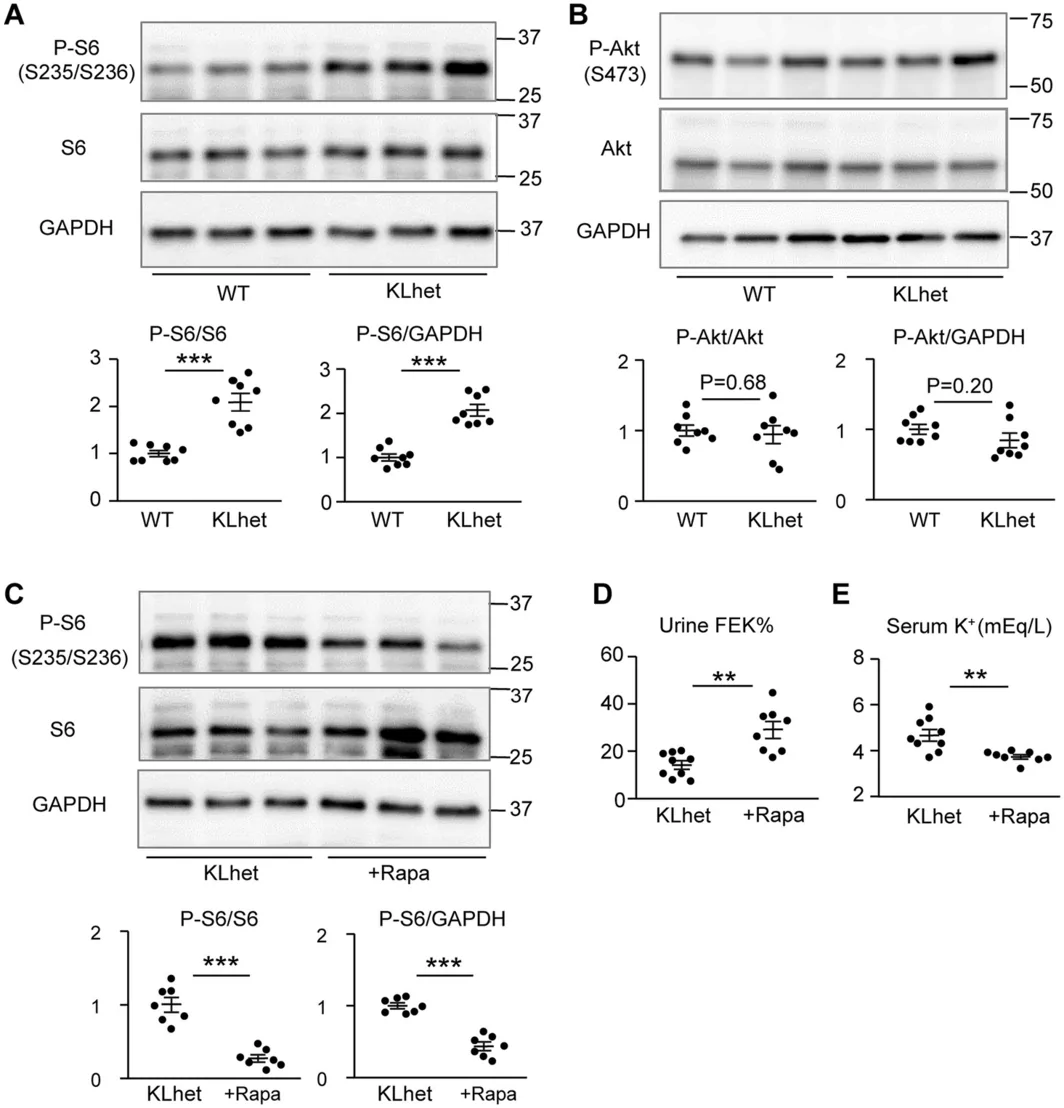
Activation of renal mTORC1 signaling and its reversal by rapamycin in KLhet mice. (A) Relative expression of phosphorylated S6 (p‐S6), normalized to total S6 and GAPDH, in whole‐kidney lysates of WT mice and KLhet mice (*n* = 8 per group). (B) Representative Western blot and quantification of phosphorylated Akt (p‐Akt), normalized to total Akt and GAPDH, in whole‐kidney lysates of WT mice and KLhet mice (*n* = 8 per group). (C) Representative Western blot and quantification of p‐S6, normalized to total S6 and GAPDH, in whole‐kidney lysates of KLhet mice and rapamycin‐treated KLhet mice (+Rapa) (*n* = 7 per group). (D) Urinary fractional excretion of potassium (FEK) in KLhet mice (*n* = 9) and KLhet + Rapa mice (*n* = 8). (E) Serum potassium (K^+^) concentrations in KLhet mice (*n* = 9) and KLhet + Rapa mice (*n* = 8). Data are presented as mean ± SEM. Welch's t test was used for comparisons between two groups. **p < 0.01, ***p < 0.001, as indicated.

Immunofluorescence staining was performed to examine the intrarenal distribution of p‐S6. Higher‐magnification images of comparable AQP2‐positive tubular segments showed that p‐S6 immunoreactivity in AQP2‐positive cells appeared more prominent in KLhet than in WT kidneys. In addition, particularly prominent p‐S6 immunoreactivity was observed in AQP2‐negative cells interspersed among AQP2‐positive cells (Figure S2A). Lower‐magnification images showed that p‐S6‐positive cells were also present in AQP2‐negative tubular segments in both WT and KLhet kidneys, with a broadly similar staining pattern between the two groups (Figure S2B). Rapamycin treatment significantly increased FEK and reduced serum potassium levels in KLhet mice (Figure 3D,E), with serum potassium decreasing from 4.7 ± 0.2 mEq/L to 3.7 ± 0.1 mEq/L (*p* = 0.004). ROMK1 abundance in the isolated plasma membrane fraction was significantly increased by rapamycin (Figure 4A). In parallel, the cleaved forms of α‐ENaC and γ‐ENaC were reduced (Figure 4B), and PAC decreased from 418.7 ± 48.9 to 220.0 ± 33.4 pg/mL (*p* = 0.005) (Figure 4C). Rapamycin did not alter renal or circulating Klotho levels (Figure S3A and Figure 4D).

**FIGURE 4:**
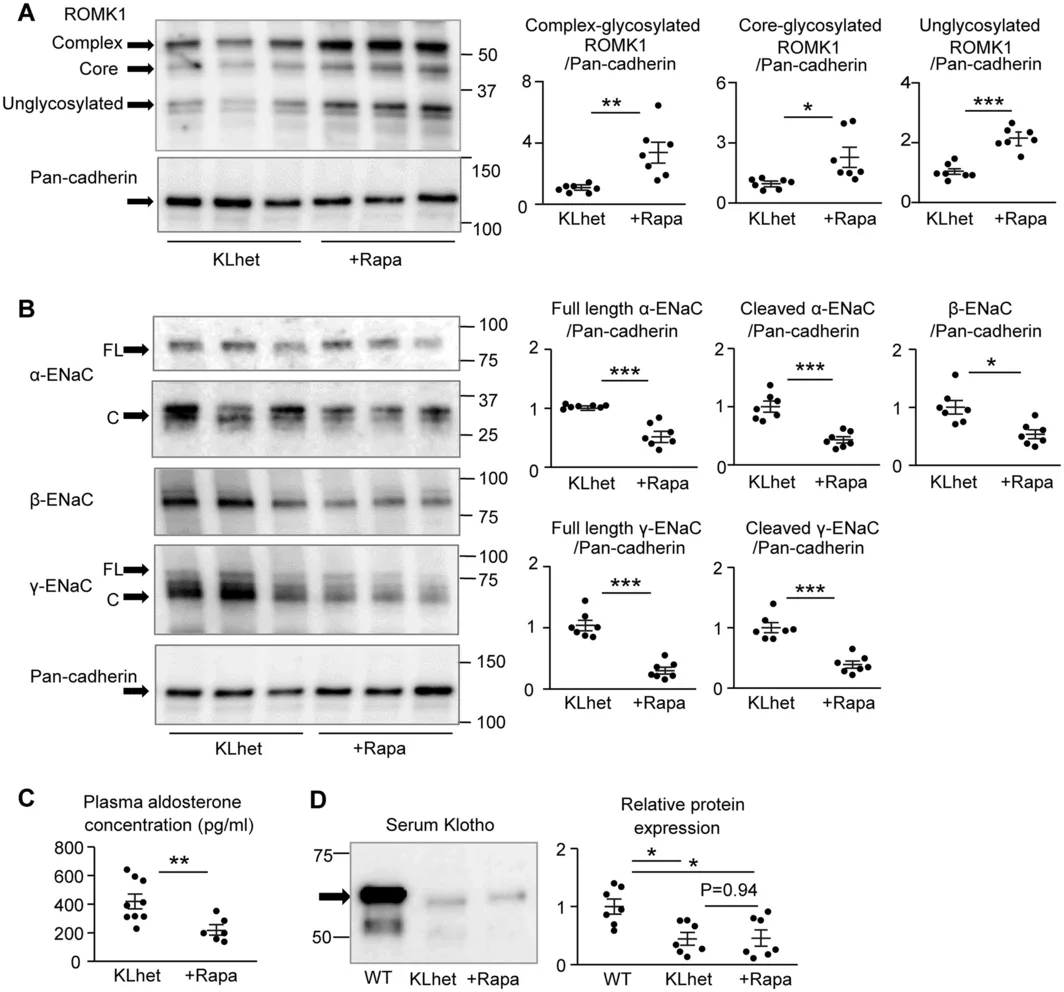
Restoration of ROMK1 and suppression of ENaC by rapamycin in KLhet mice. (A) Representative Western blot and quantification of ROMK1 protein expression in renal plasma membrane fractions of KLhet mice and rapamycin‐treated KLhet mice (+Rapa) (*n* = 7 per group). (B) Representative Western blot and quantification of α‐, β‐, and γ‐ENaC in renal plasma membrane fractions of KLhet mice and KLhet + Rapa mice. Full‐length (FL) and cleaved (C) forms of α‐ and γ‐ENaC are shown. Pan‐cadherin was used as a loading control (*n* = 7 per group). (C) Plasma aldosterone concentrations in KLhet mice (*n* = 9) and KLhet + Rapa mice (*n* = 6). (D) Representative Western blot and quantification of serum Klotho levels in WT mice, KLhet mice, and KLhet + Rapa mice (*n* = 7 per group). No significant difference in Klotho levels was observed between KLhet and KLhet + Rapa mice. Data are presented as mean ± SEM. Welch's *t*‐test was used for comparisons between two groups (A–C), and one‐way ANOVA followed by Holm–Bonferroni‐adjusted pairwise comparisons was used for comparisons among three groups (D). *p < 0.05, **p < 0.01, ***p < 0.001, as indicated.

To examine whether rapamycin exerted similar effects on renal K^+^ handling in WT mice, WT mice were treated with rapamycin using the same protocol. Rapamycin significantly reduced renal p‐S6 levels but did not alter FEK (32.1% ± 1.9% vs. 31.9% ± 2.2%; *p* = 0.96), while serum potassium showed a nonsignificant trend toward a decrease (4.0 ± 0.1 vs. 3.7 ± 0.1 mEq/L; *p* = 0.07) (Figure S4A,B). Rapamycin modestly altered some components of distal K^+^ transport, including an increase in core‐glycosylated ROMK1 and a decrease in full‐length γ‐ENaC, without significant changes in PAC or renal or circulating Klotho levels (Figures S4C and S5). Thus, although rapamycin modulated some components of distal K^+^ transport in WT mice, it did not increase FEK, in contrast to the marked improvement in renal K^+^ handling observed in KLhet mice.

### 3.4 Klotho Supplementation Restores ROMK1 Expression and Potassium Homeostasis in KLhet Mice

Klotho supplementation increased urinary FEK (Figure 5A) and reduced serum potassium levels in KLhet mice (Figure 5B), with serum potassium decreasing from 4.7 ± 0.2 to 3.7 ± 0.1 mEq/L (*p* = 0.004). These changes were accompanied by increased renal and circulating Klotho protein levels (Figure S3B and Figure 5C) and reduced renal p‐S6 expression (Figure 5D).

**FIGURE 5:**
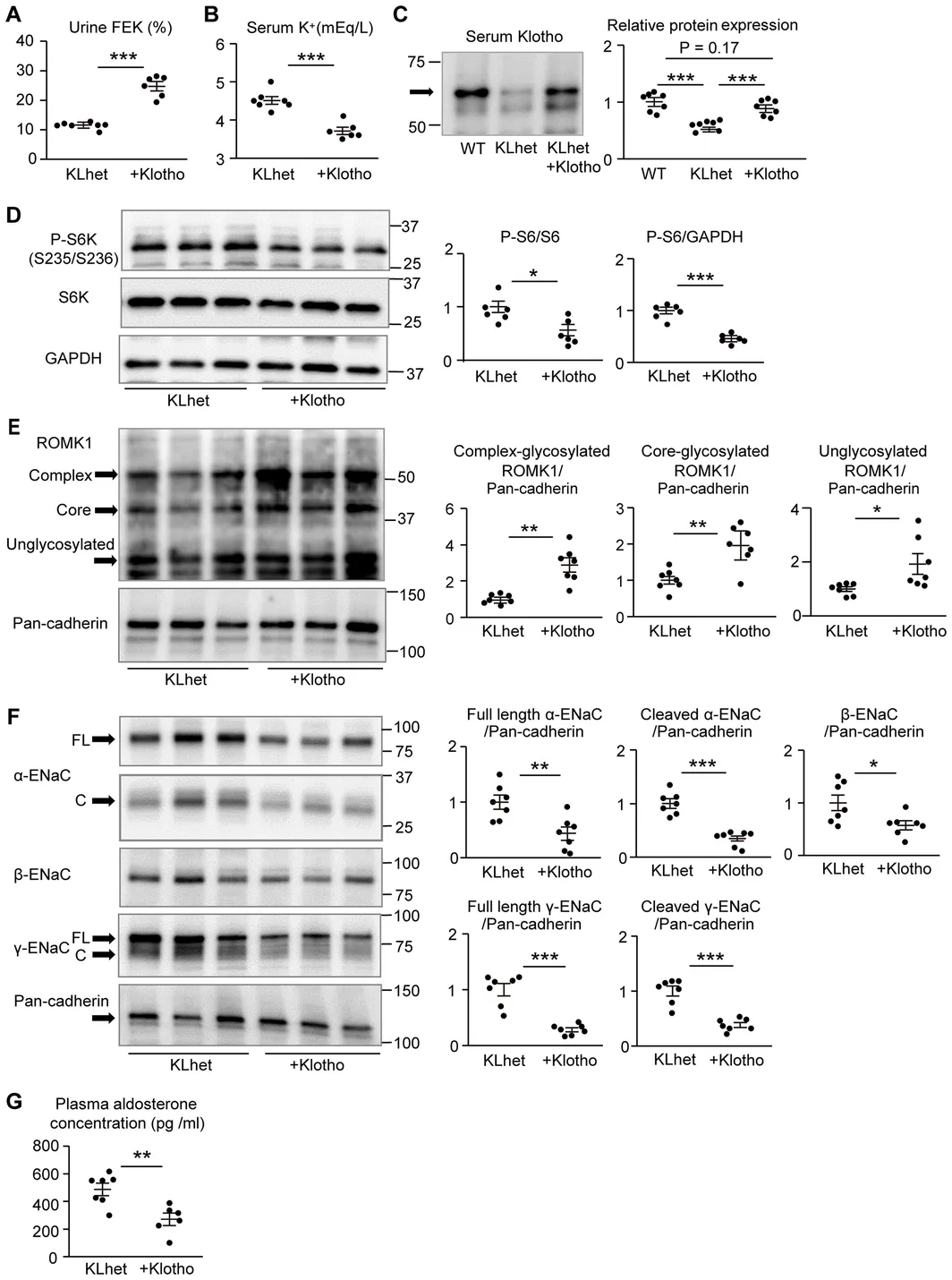
Klotho supplementation restores potassium homeostasis and suppresses mTORC1 signaling in KLhet mice. (A) Urinary fractional excretion of potassium (FEK) in KLhet mice (*n* = 7) and KLhet mice treated with Klotho supplementation (+Klotho) (*n* = 6). (B) Serum potassium (K^+^) concentrations in KLhet (*n* = 7) and KLhet + Klotho mice (*n* = 6). (C) Representative Western blot and quantification of serum Klotho protein expression in WT, KLhet, and KLhet + Klotho mice (*n* = 7 per group). No significant difference was observed between WT and KLhet + Klotho groups. (D) Representative Western blot and quantification of phosphorylated S6 (p‐S6; Ser235/236), normalized to total S6 and GAPDH, in whole‐kidney lysates from KLhet and KLhet + Klotho mice (*n* = 6 per group). (E) Representative Western blot and quantification of ROMK1 protein expression in renal plasma membrane fractions from KLhet and KLhet + Klotho mice. Pan‐cadherin was used as a loading control (*n* = 7 per group). (F) Representative Western blot and quantification of α‐, β‐, and γ‐ENaC in renal plasma membrane fractions from KLhet and KLhet + Klotho mice. Pan‐cadherin was used as a loading control (*n* = 7 per group). (G) Plasma aldosterone concentrations in KLhet (*n* = 7) and KLhet + Klotho (*n* = 6). (A–G) Control KLhet mice were injected intraperitoneally with 50 μL of normal saline. Data are presented as mean ± SEM. Welch's *t*‐test was used for comparisons between two groups, and one‐way ANOVA followed by Holm–Bonferroni‐adjusted pairwise comparisons was used for comparisons among three groups. *p < 0.05, **p < 0.01, ***p < 0.001, as indicated.

The complex‐glycosylated, core‐glycosylated, and unglycosylated forms of ROMK1 in the isolated plasma membrane fraction were all significantly increased by Klotho supplementation (Figure 5E). In parallel, the cleaved forms of α‐ and γ‐ENaC were reduced (Figure 5F), and PAC was significantly decreased (from 486.7 ± 42.1 to 270.5 ± 41.2 pg/mL, *p* = 0.004; Figure 5G).

### 3.5 Effects of Rapamycin and Klotho Supplementation on ROMK Colocalization With AQP2 in the Distal Nephron

ROMK is expressed in multiple nephron segments, including the thick ascending limb (TAL), DCT2, CNT, and CCD, with distinct roles in apical K^+^ recycling in the TAL and K^+^ secretion in distal nephron segments. Immunofluorescence analysis showed ROMK immunoreactivity in both cytoplasmic and apical regions of AQP2‐positive tubular cells. In WT kidneys, ROMK immunoreactivity showed prominent overlap with apically localized AQP2. This apical overlap appeared reduced in KLhet kidneys but more prominent following treatment with either rapamycin or Klotho supplementation (Figure 6A,B).

**FIGURE 6:**
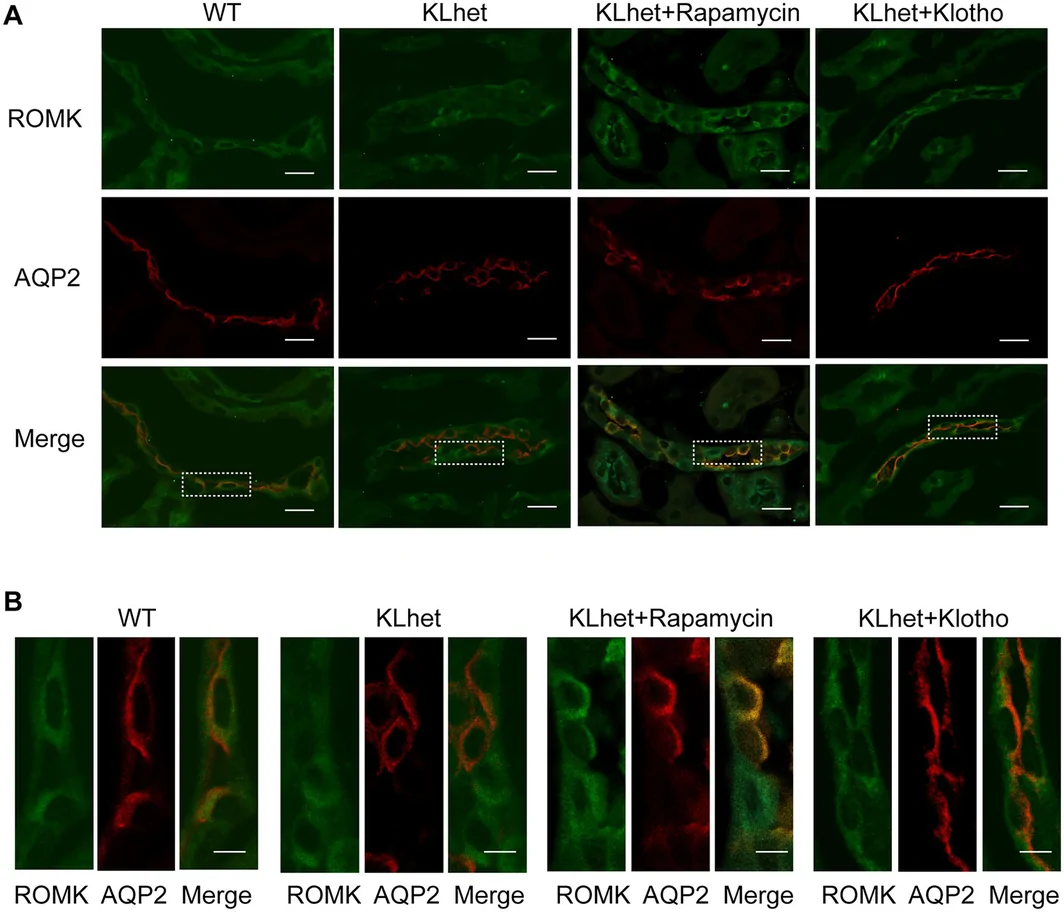
Reduced apical ROMK localization in AQP2‐positive principal cells of KLhet mice and its restoration by rapamycin or Klotho supplementation. (A) Representative immunofluorescence images of ROMK (green) and AQP2 (red) in WT, KLhet, KLhet + rapamycin, and KLhet + Klotho mice. Images were acquired and displayed at the same magnification across all groups. Dotted boxes indicate the regions enlarged in (B). Scale bars, 20 μm. (B) Enlarged views of the boxed regions shown in (A), displaying ROMK, AQP2, and merged channels. All enlarged views are shown at the same magnification across groups. Scale bars, 5 μm.

To assess segment specificity, double immunofluorescence staining for ROMK and Tamm–Horsfall protein (THP) was performed. In THP‐positive TAL segments, ROMK immunoreactivity was observed along the apical aspect in both WT and KLhet kidneys, with no obvious difference in the staining pattern between the groups (Figure S6).

Given the importance of Na^+^ delivery from the DCT for downstream K^+^ secretion, we also examined NCC expression and phosphorylation. pT53‐NCC abundance in renal plasma membrane fractions was significantly increased in KLhet mice compared with WT mice, whereas total NCC showed a trend toward an increase that did not reach statistical significance (*p* = 0.07; Figure S7A). Rapamycin treatment did not significantly alter pT53‐NCC (*p* = 0.09) or total NCC abundance (*p* = 0.41) in KLhet mice (Figure S7B).

We further examined alternative potassium secretory pathways. Western blot analysis of plasma membrane fractions showed no significant difference in BKα protein abundance between WT and KLhet mice (*p* = 0.52; Figure S8A). Immunofluorescence staining showed a broadly similar intrarenal distribution of BKα in WT and KLhet kidneys (Figure S8B).

### 3.6 Rapamycin or Klotho Supplementation Suppresses Adrenal Cyp11b2 Expression in KLhet Mice

Plasma aldosterone concentrations were elevated in KLhet mice compared with WT controls (Figure 2C). This was accompanied by increased adrenal *Cyp11b2* mRNA expression, which encodes aldosterone synthase (Figure 7A), whereas *Cyp11b1* mRNA expression did not differ between groups. Klotho supplementation or rapamycin treatment reduced *Cyp11b2* expression in KLhet mice, while *Cyp11b1* expression remained unchanged (Figure 7B).

**FIGURE 7:**
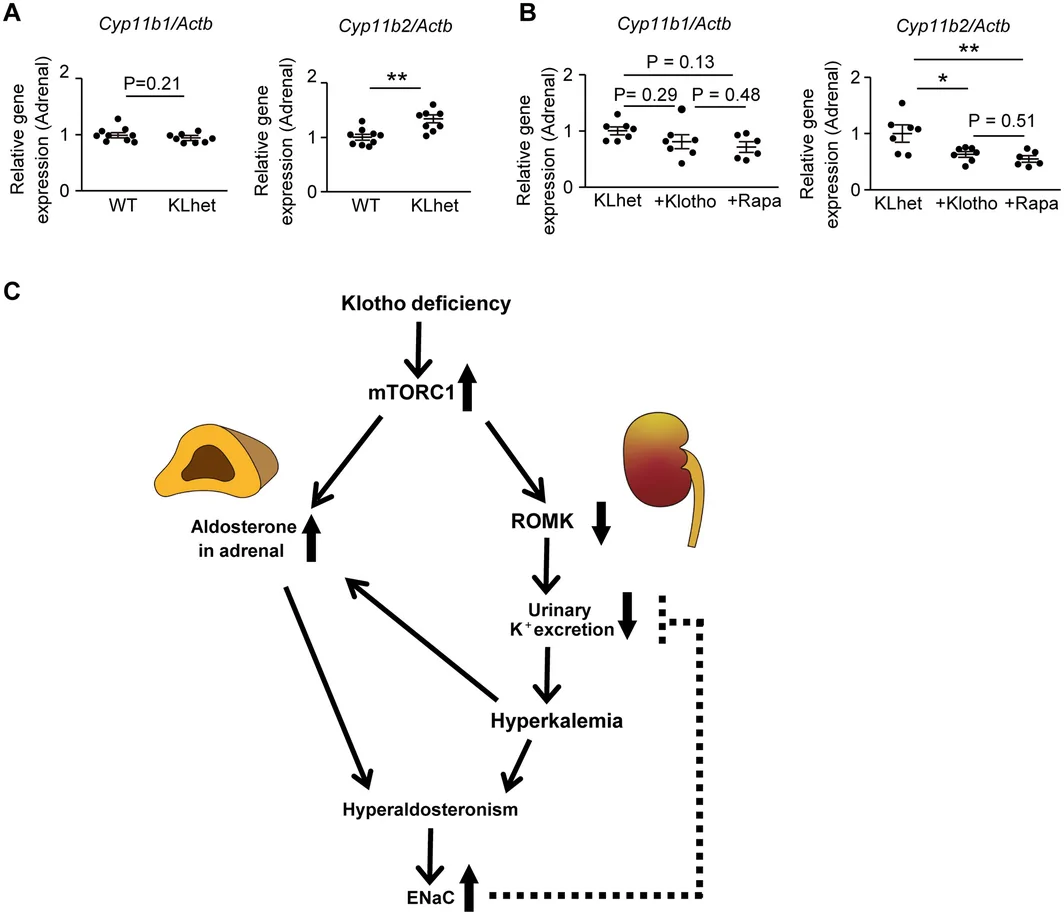
Regulation of adrenal Cyp11b2 expression and proposed mechanism of hyperkalemia in KLhet mice. (A) Relative adrenal mRNA expression of *Cyp11b1* and *Cyp11b2* in WT mice (*n* = 9) and KLhet mice (*n* = 8). (B) Relative adrenal mRNA expression of *Cyp11b1* and *Cyp11b2* in KLhet (*n* = 7), KLhet mice treated with Klotho supplementation (+Klotho, *n* = 7), and KLhet mice treated with rapamycin (+Rapa, *n* = 6). Data are presented as mean ± SEM. Welch's *t*‐test was used for comparisons between two groups (A), and one‐way ANOVA followed by Holm–Bonferroni‐adjusted pairwise comparisons was used for comparisons among three groups (B). *p < 0.05, **p < 0.01. (C) Proposed mechanism of Klotho deficiency–induced hyperkalemia. Under conditions of Klotho deficiency, activation of renal mTORC1 reduces the plasma membrane abundance of ROMK, leading to impaired urinary potassium excretion and hyperkalemia. ENaC activation, likely mediated by increased aldosterone production in response to hyperkalemia, partially compensates for impaired potassium excretion due to decreased apical ROMK. Bold arrows indicate stimulation (upward) or inhibition (downward), and the dotted line indicates partial compensation.

## 4 Discussion

In this study, we demonstrated that Klotho deficiency activates renal mTORC1 signaling, leading to reduced plasma membrane expression of ROMK1, impaired urinary potassium excretion, and mild hyperkalemia in mice. Both rapamycin and Klotho supplementation normalized these abnormalities, suggesting that mTORC1 activation mediates the link between Klotho deficiency and disordered potassium homeostasis. In addition, aldosterone synthesis was increased in KLhet mice, likely contributing to compensatory activation of ENaC in response to hyperkalemia caused by impaired potassium excretion (Figure 7C).

Given that hyperkalemia is a potent stimulus for aldosterone production, the suppression of aldosterone synthesis observed after rapamycin treatment and Klotho supplementation is likely secondary to normalization of serum potassium levels. However, direct effects of rapamycin or Klotho supplementation on adrenal aldosterone synthesis cannot be excluded. This interpretation is supported by previous studies suggesting that mTORC1 signaling may contribute to enhanced adrenal steroidogenesis [30]. In addition, a prior in vitro study showed that silencing of Klotho upregulated *CYP11b2* expression in human adrenocortical cells [31]. These findings suggest that mTORC1 activation or Klotho deficiency may promote aldosterone synthesis via a hyperkalemia‐independent pathway.

In humans, circulating Klotho levels decline with aging, reaching approximately half of their peak levels by the seventh decade of life [32, 33]. Similarly, both aged mice and KLhet mice exhibit comparable reductions in circulating Klotho levels [11], supporting the relevance of this model to age‐related Klotho deficiency.

Accumulating evidence indicates that the pleiotropic actions of circulating Klotho are closely linked to mTORC1 activation under conditions of Klotho deficiency [21, 22]. For example, mesenchymal stem cells derived from Klotho‐deficient mice exhibit mTORC1 activation and abnormal proliferation, both of which are reversed by soluble Klotho [21]. Consistent with these findings, we observed increased renal mTORC1 activity in KLhet mice, which was associated with reduced ROMK1 expression and hyperkalemia. Importantly, rapamycin did not restore renal Klotho expression, suggesting that Klotho acts upstream of mTORC1 signaling. Interestingly, immunofluorescence analysis showed that p‐S6 immunoreactivity in AQP2‐positive cells appeared more prominent in KLhet than in WT kidneys. In addition, particularly prominent p‐S6 immunoreactivity was observed in AQP2‐negative cells interspersed among AQP2‐positive cells. A recent preprint similarly reported preferential p‐S6 expression in AQP2‐negative collecting duct cells and further suggested that genetic mTORC1 activation in principal cells may promote acquisition of intercalated‐cell characteristics [34]. Although these findings raise the possibility that mTORC1 signaling may influence collecting duct cell states, further studies using cell‐type‐specific markers and lineage tracing are required to determine the significance of this staining pattern in KLhet mice.

With respect to the functional role of mTORC1 signaling in renal K^+^ handling, rapamycin effectively suppressed renal mTORC1/S6 signaling in WT mice but did not increase FEK, suggesting that mTORC1 inhibition per se does not necessarily promote renal K^+^ excretion under basal conditions. The more pronounced effect of rapamycin on K^+^ excretion in KLhet mice may therefore reflect the underlying enhancement of mTORC1/S6 signaling associated with Klotho haploinsufficiency. Given that both Klotho deficiency and mTORC1 activation are implicated in aging‐related tissue dysfunction, targeting mTORC1 signaling may represent a potential therapeutic strategy for age‐associated electrolyte imbalance and renal dysfunction [35].

Previous in vitro studies have shown that Klotho enhances ROMK1 membrane expression by modifying glycan structures and inhibiting channel endocytosis [15]. In vivo, soluble Klotho administration increases urinary potassium excretion in rats [15]. In the present study, Klotho deficiency was associated with activation of renal mTORC1 signaling and reduced ROMK1 abundance in plasma membrane fractions, accompanied by impaired urinary potassium excretion and hyperkalemia. Although the precise trafficking mechanisms of ROMK were not directly assessed, these findings suggest that mTORC1 activation contributes to reduced membrane availability of ROMK1 in Klotho‐deficient states.

ROMK channels in the distal nephron play a central role in potassium excretion under physiological conditions, whereas BKα channels contribute to flow‐dependent potassium secretion [36]. In the present study, BKα protein abundance in renal plasma membrane fractions was unchanged between WT and KLhet mice, whereas apical ROMK staining was reduced in AQP2‐positive distal nephron segments and was associated with hyperkalemia in KLhet mice. Although our findings do not exclude alterations in BK channel activity or apical trafficking, they suggest that changes in BKα abundance in the renal plasma membrane fraction are unlikely to account for the altered renal K^+^ handling in KLhet mice. Taken together with the reduced plasma membrane abundance of ROMK1 in KLhet mice, these findings support the concept that ROMK plays a central role in distal potassium secretion and contributes to potassium homeostasis under physiological conditions.

Although ROMK is also expressed in the TAL, where it supports NKCC2‐mediated NaCl reabsorption through apical K^+^ recycling [37], our immunofluorescence analysis did not demonstrate reduced ROMK expression in TAL segments. Together with the observed reduction in AQP2‐positive distal nephron segments, these findings suggest that ROMK downregulation occurs predominantly in the distal nephron rather than in the TAL. Because immunoblot analysis was performed using whole kidney plasma membrane fractions, segment‐specific differences could not be resolved; therefore, immunofluorescence with segment‐specific markers and a pan‐ROMK antibody was used to assess ROMK localization in distal nephron segments. This complementary approach allowed us to assess overall plasma membrane abundance and segment‐specific localization of ROMK.

Interestingly, pT53‐NCC abundance was increased in KLhet mice despite hyperkalemia, a condition that would normally suppress NCC phosphorylation and increase Na^+^ delivery to downstream K^+^‐secretory segments [38, 39]. This unexpected finding raises the possibility that Klotho haploinsufficiency may interfere with the physiological K^+^‐dependent regulation of NCC. Enhanced NCC activation could reduce distal Na^+^ delivery and thereby further contribute to impaired K^+^ secretion in KLhet mice. However, rapamycin did not significantly reduce pT53‐NCC abundance despite improving urinary K^+^ excretion and hyperkalemia. Thus, although altered NCC regulation may contribute to the potassium‐handling phenotype of KLhet mice, the beneficial effect of mTORC1 inhibition on K^+^ homeostasis is unlikely to be mediated primarily through suppression of NCC. The mechanism underlying the unexpected increase in NCC phosphorylation in KLhet mice remains to be determined.

Elevated aldosterone levels likely promote ENaC activation by generating a lumen‐negative transepithelial voltage, thereby serving as a compensatory mechanism to counteract impaired potassium excretion resulting from reduced plasma membrane abundance of ROMK1. This interpretation is supported by the observation that severe hyperkalemia did not develop until the ENaC inhibitor amiloride was administered. This concept is further supported by clinical observations indicating that older adults, who are more susceptible to hyperkalemia, may remain clinically stable due to compensatory mechanisms, but this vulnerability becomes unmasked when ENaC activity is suppressed by pharmacological interventions such as mineralocorticoid receptor antagonists [40, 41].

Interestingly, the present finding that KLhet mice exhibit increased ENaC activity despite reduced ROMK‐mediated potassium excretion appears to challenge the classical paradigm in which ENaC activation promotes potassium secretion. ENaC and ROMK are colocalized in principal cells, and under basal conditions, DCT2 and early CNT exhibit substantially greater ENaC and ROMK transport capacities than the CD [42, 43]. However, ENaC activity in early distal segments (DCT2/early CNT) is largely aldosterone‐independent, whereas aldosterone responsiveness is greater in the late CNT/CD [44]. Accordingly, Klotho deficiency may enhance ENaC activity via hyperaldosteronism predominantly in the late CNT and CD, whereas reduced ROMK abundance may impair K^+^ secretion in DCT2, CNT, and CD despite this compensatory ENaC activation. Given this disproportionate regulation of ENaC and ROMK, direct electrophysiological evaluation will be necessary to determine channel activities in distinct tubular segments of KLhet mice.

Klotho insufficiency can alter mineral metabolism, including phosphate and calcitriol homeostasis, which may secondarily influence the RAAS. However, in the present study, serum calcium, phosphate, and 1,25‐(OH)_2_D_3_ levels did not differ significantly between WT and KLhet mice (Figure S1). In addition, we previously found no significant difference in serum FGF23 levels between WT and KLhet mice at the same age [11]. These findings suggest that overt alterations in the Klotho–FGF23–phosphate–vitamin D axis are unlikely to account for the increased aldosterone levels observed in KLhet mice. Nevertheless, subtle effects of Klotho deficiency on mineral metabolism or RAAS regulation that are not reflected by circulating levels cannot be completely excluded.

This study has several limitations. First, only male mice were examined. Given known sex differences in renal electrolyte handling, future studies are needed to determine whether these findings apply to female mice. Second, WT and KLhet mice were not littermates but were separately bred animals with the same C57BL/6JJcl background. Although age‐matched C57BL/6JJcl mice were used as controls, we cannot completely exclude potential confounding effects related to differences in breeding conditions. Third, we did not directly assess adrenal mTORC1 activation. Although rapamycin reduced *Cyp11b2* expression, suggesting involvement of adrenal mTORC1 signaling, direct biochemical evidence is lacking. In addition, enhanced adrenal mTORC1 signaling may be relevant to conditions such as age‐related autonomous aldosteronism, which is characterized by impaired aldosterone suppression despite high salt intake [45, 46]; however, further studies are needed to establish this link.

## 5 Conclusion

Klotho deficiency impairs urinary potassium excretion and leads to hyperkalemia, accompanied by renal mTORC1 activation and reduced plasma membrane abundance of ROMK1 in the distal nephron. This renal defect, together with adrenal‐driven hyperaldosteronism and ENaC activation, may represent a compensatory response to maintain potassium balance. Both Klotho supplementation and mTORC1 inhibition reversed these abnormalities. Collectively, these findings suggest that the Klotho–mTORC1–ROMK axis represents a key pathway linking Klotho deficiency, a feature of aging, to dysregulation of renal potassium homeostasis and may provide a potential therapeutic target for preventing electrolyte imbalance in aging.

## Author Contributions

**Wakako Kawarazaki:** conceptualization, investigation, funding acquisition, writing – original draft, methodology, visualization, writing – review and editing, project administration, data curation, resources, formal analysis. **Nobuhiro Ayuzawa:** writing – review and editing, resources. **Mitsuhiro Nishimoto:** writing – review and editing, resources. **Daigoro Hirohama:** writing – review and editing, resources. **Kohei Ueda:** writing – review and editing, resources. **Takeshi Marumo:** writing – review and editing, resources. **Toshiro Fujita:** conceptualization, funding acquisition, methodology, writing – review and editing, resources.

## Funding

This work was supported by the Japan Society for the Promotion of Science KAKENHI (Grant numbers: 23K07680 and 26K11205 to W.K. and 15H05788 to T.F.); the SENSHIN Medical Research Foundation; the Takeda Science Foundation; the Astellas Foundation for Research on Metabolic Disorders; a Basic Research Grant from the Japanese Society of Hypertension; an Aging and Geriatrics Research Grant from the Japan Geriatrics Society; the Naito Foundation; and the Harmonic Ito Foundation.

## Conflicts of Interest

Wakako Kawarazaki reports research support from JSPS KAKENHI (23K07680 and 26K11205), the Takeda Science Foundation, the Astellas Foundation for Research on Metabolic Disorders, the SENSHIN Medical Research Foundation, the Naito Foundation, the Harmonic Ito Foundation, the Basic Research Grant of the Japanese Society of Hypertension, and the Aging and Geriatrics Research Grant of the Japan Geriatrics Society. All funding was paid to her institution. Toshiro Fujita reports support from JSPS KAKENHI (15H05788). The remaining authors declare no conflicts of interest.

## Supporting information


**Table S1:** Antibodies used for western blot (WB) and immunofluorescence (IF). The table lists the primary antibodies used in this study, including species, dilution factors for WB and IF, suppliers, and catalog numbers.
**Figure S1:** Serum calcium, phosphate, and 1,25‐dihydroxyvitamin D_3_ levels in WT and KLhet mice. Serum calcium (*n* = 9 per group), phosphate (*n* = 9 per group), and 1,25‐dihydroxyvitamin D_3_ [1,25‐(OH)_2_D_3_] concentrations (*n* = 5 per group) in WT and KLhet mice. Data are presented as mean ± SEM. Welch's *t*‐test was used for comparisons between two groups.
**Figure S2:** Intrarenal distribution of p‐S6 in WT and KLhet kidneys. (A) Representative higher‐magnification images of double immunofluorescence staining for phosphorylated S6 (p‐S6; green) and aquaporin‐2 (AQP2; red) in kidneys from WT and KLhet mice. Within AQP2‐positive tubular segments, p‐S6 immunoreactivity was observed in AQP2‐positive cells and was particularly prominent in AQP2‐negative cells. (B) Representative lower‐magnification merged images showing the heterogeneous tubular distribution of p‐S6 immunoreactivity in WT and KLhet kidneys. p‐S6 immunoreactivity was also observed in AQP2‐negative tubular segments in both groups. Scale bars, 20 μm.
**Figure S3:** Renal Klotho protein expression is not altered by rapamycin treatment but is restored by Klotho supplementation in KLhet mice. (A) Representative Western blot and quantification of renal Klotho protein expression in whole‐kidney lysates from KLhet mice and KLhet mice treated with rapamycin (+Rapa). GAPDH was used as a loading control. No significant difference was observed between groups (*n* = 7 per group; *p* = 0.72). (B) Representative Western blot and quantification of renal Klotho protein expression in whole‐kidney lysates from KLhet mice and KLhet mice treated with Klotho supplementation (+Klotho). Klotho expression was significantly increased following supplementation (*n* = 6 per group; **p < 0.01). Data are presented as mean ± SEM. Welch's *t*‐test was used for comparisons between two groups. **p < 0.01. N.S.
**Figure S4:** Effects of rapamycin on renal mTORC1/S6 signaling, potassium handling, and ROMK1 expression in WT mice. (A) Representative Western blot and quantification of phosphorylated S6 (pS6) expression in whole‐kidney lysates from WT and rapamycin‐treated WT (WT + Rapa) mice. pS6 levels were normalized to total S6 and GAPDH (*n* = 7 per group). (B) Fractional urinary potassium excretion (FEK) and serum potassium (K+) concentrations in WT and WT + Rapa mice (*n* = 7 per group). (C) Representative Western blot and quantification of ROMK1 protein expression in renal plasma membrane fractions. Complex‐glycosylated, core‐glycosylated, and unglycosylated forms are shown (*n* = 6 per group). Data are presented as mean ± SEM. Welch's t test was used for comparisons between two groups. *p < 0.05, **p < 0.01, as indicated. Rapa, rapamycin.
**Figure S5:** Effects of rapamycin on ENaC expression, plasma aldosterone, and Klotho levels in WT mice. (A) Representative Western blot and quantification of α‐, β‐, and γ‐subunits of ENaC in renal plasma membrane fractions from WT and rapamycin‐treated WT (WT + Rapa) mice. Full‐length (FL) and cleaved (C) forms of α‐ENaC and γ‐ENaC, and full‐length β‐ENaC are shown (*n* = 6 per group). (B) Plasma aldosterone concentrations in WT and WT + Rapa mice (*n* = 7 per group). (C) Representative Western blot and quantification of renal Klotho protein expression in whole‐kidney lysates from WT and WT + Rapa mice (*n* = 7 per group). (D) Serum Klotho concentrations in WT and WT + Rapa mice (*n* = 7 per group). Data are presented as mean ± SEM. Welch's t test was used for comparisons between two groups. *p < 0.05, as indicated. C, cleaved; FL, full‐length; Rapa, rapamycin.
**Figure S6:** ROMK expression in Tamm–Horsfall protein (THP)‐positive thick ascending limb (TAL) segments is preserved in KLhet mice. Representative immunofluorescence staining for ROMK (green) and THP (red) in kidney sections from WT and KLhet mice. Merged images are shown in the third row. ROMK expression in THP‐positive TAL segments was comparable between WT and KLhet mice, with no apparent reduction in KLhet mice. Magnified images (bottom row) show higher‐magnification views of the indicated regions. Scale bars, 20 μm.
**Figure S7:** NCC expression and phosphorylation in KLhet mice and the effects of rapamycin.
(A) Representative Western blot and quantification of phosphorylated NCC at Thr53 (pT53‐NCC) and total NCC in renal plasma membrane fractions from WT and KLhet mice. pT53‐NCC and total NCC were normalized to pan‐cadherin (*n* = 7 per group). (B) Representative Western blot and quantification of pT53‐NCC and total NCC in renal plasma membrane fractions from KLhet and rapamycin‐treated KLhet (KLhet + Rapa) mice. pT53‐NCC and total NCC were normalized to pan‐cadherin (*n* = 6 per group).
**Figure S8:** Renal BKα expression in WT and KLhet mice. (A) Representative Western blot and quantification of BKα protein expression in renal plasma membrane fractions from WT and KLhet mice. BKα expression was normalized to pan‐cadherin (*n* = 8 per group). (B) Double immunofluorescence staining for BKα (green) and aquaporin‐2 (AQP2; red) in kidneys from WT and KLhet mice. Representative images and magnified views are shown. Scale bars, 20 μm. Data are presented as mean ± SEM. Welch's *t*‐test was used for comparisons between two groups.

## Data Availability

The datasets generated and/or analyzed during the current study are included in this published article. Additional materials, including protocols and animal models, are available from the corresponding author upon reasonable request.
