# Supplementary material for: Klotho Deficiency Impairs Distal Potassium Homeostasis Through mTORC1‐Dependent Suppression of ROMK
Source: Acta Physiol (Oxf). 2026 Oct 4;242(11):e70325. doi: 10.1111/apha.70325 (PMC13635471; doi:10.1111/apha.70325)
Supplement: Supplementary file 1 [file APHA-242-e70325-s001.pdf]

Supplementary Table 1.

Antibodies used for western blot (WB) and immunofluorescence (IF).

The table lists the primary antibodies used in this study, including species, dilution factors for WB and IF, suppliers, and catalog numbers.

Name	Species	Dilution for WB	Dilution for IF	Supplier	Cat. Number
ROMK1	Rabbit	1:4,000	–	Alomone Labs	APC-001
Pan-ROMK	Rabbit	–	1:100	Sigma-Aldrich	SAB4501630
BKα	Rabbit	1:4,000	1:200	Alomone Labs	APC-107
α-ENaC	Rabbit	1:50,000	–	Gift (J. Loffing)	–
β-ENaC	Rabbit	1:80,000	–	Gift (J. Loffing)	–
γ-ENaC	Rabbit	1:10,000	–	Gift (J. Loffing)	–
Phospho-NCC (Thr53)	Rabbit	1:10,000	–	Gift (J. Loffing)	–
NCC	Rabbit	1:3,000	–	Millipore	AB3553
AQP2	Goat	–	1:200	Santa Cruz Biotechnology	sc-9882
THP (uromodulin)	Goat	–	1:200	Santa Cruz Biotechnology	sc-19554
Phospho-S6 (Ser235/236)	Rabbit	1:3,000	1:200	Cell Signaling Technology	#2211
S6	Rabbit	1:3,000	–	Cell Signaling Technology	#2217
Phospho-Akt (Ser473)	Rabbit	1:3,000	–	Cell Signaling Technology	#9271
Akt	Rabbit	1:3,000	–	Cell Signaling Technology	#9272
Pan-cadherin	Rabbit	1:3,000	–	Sigma-Aldrich	C3678
GAPDH	Mouse	1:5,000	–	Abcam	ab8245
Klotho	Rabbit	1:4,000	–	Abcam	ab154163

Supplementary Figure 1

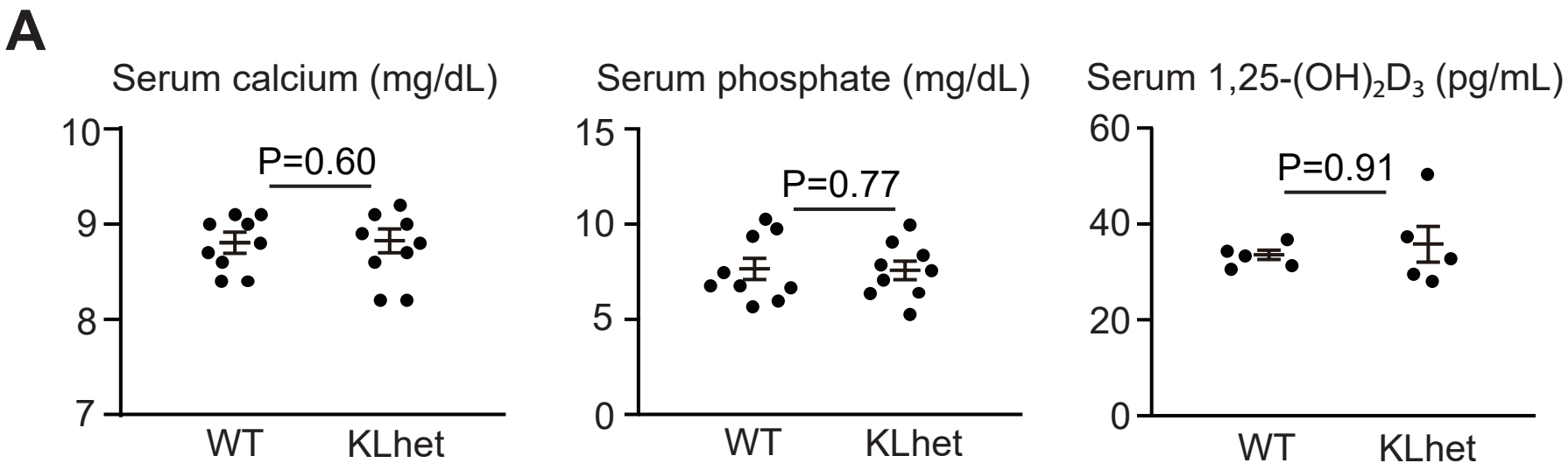

Supplementary Figure 1. Serum calcium, phosphate, and 1,25-dihydroxy vitamin D_3 levels in WT and KLhet mice.

(A) Serum calcium (*n* = 9 per group), phosphate (*n* = 9 per group), and 1,25-dihydroxyvitamin D_3 [1,25-$(OH)_2D_3$] concentrations (*n* = 5 per group) in WT and KLhet mice. Data are presented as mean ± SEM. Welch's t test was used for comparisons between two groups.

Supplementary Figure 2

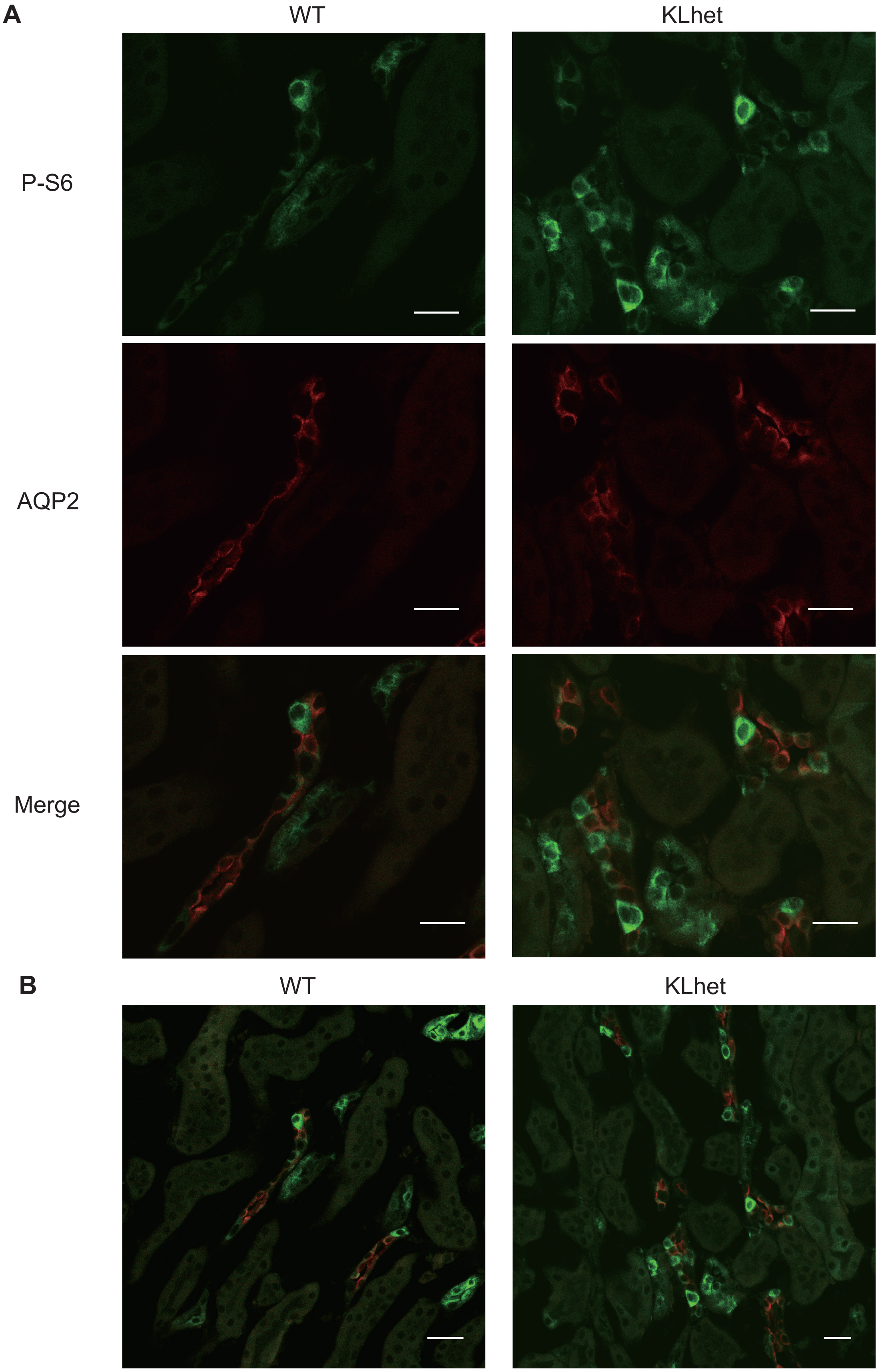

Supplementary Figure 2. Intrarenal distribution of p-S6 in WT and KLhet kidneys.

(A) Representative higher-magnification images of double immunofluorescence staining for phosphorylated S6 (p-S6; green) and aquaporin-2 (AQP2; red) in kidneys from WT and KLhet mice. Within AQP2-positive tubular segments, p-S6 immunoreactivity was observed in AQP2-positive cells and was particularly prominent in AQP2-negative cells.

(B) Representative lower-magnification merged images showing the heterogeneous tubular distribution of p-S6 immunoreactivity in WT and KLhet kidneys. p-S6 immunoreactivity was also observed in AQP2-negative tubular segments in both groups. Scale bars, 20 μm.

Supplementary Figure 3

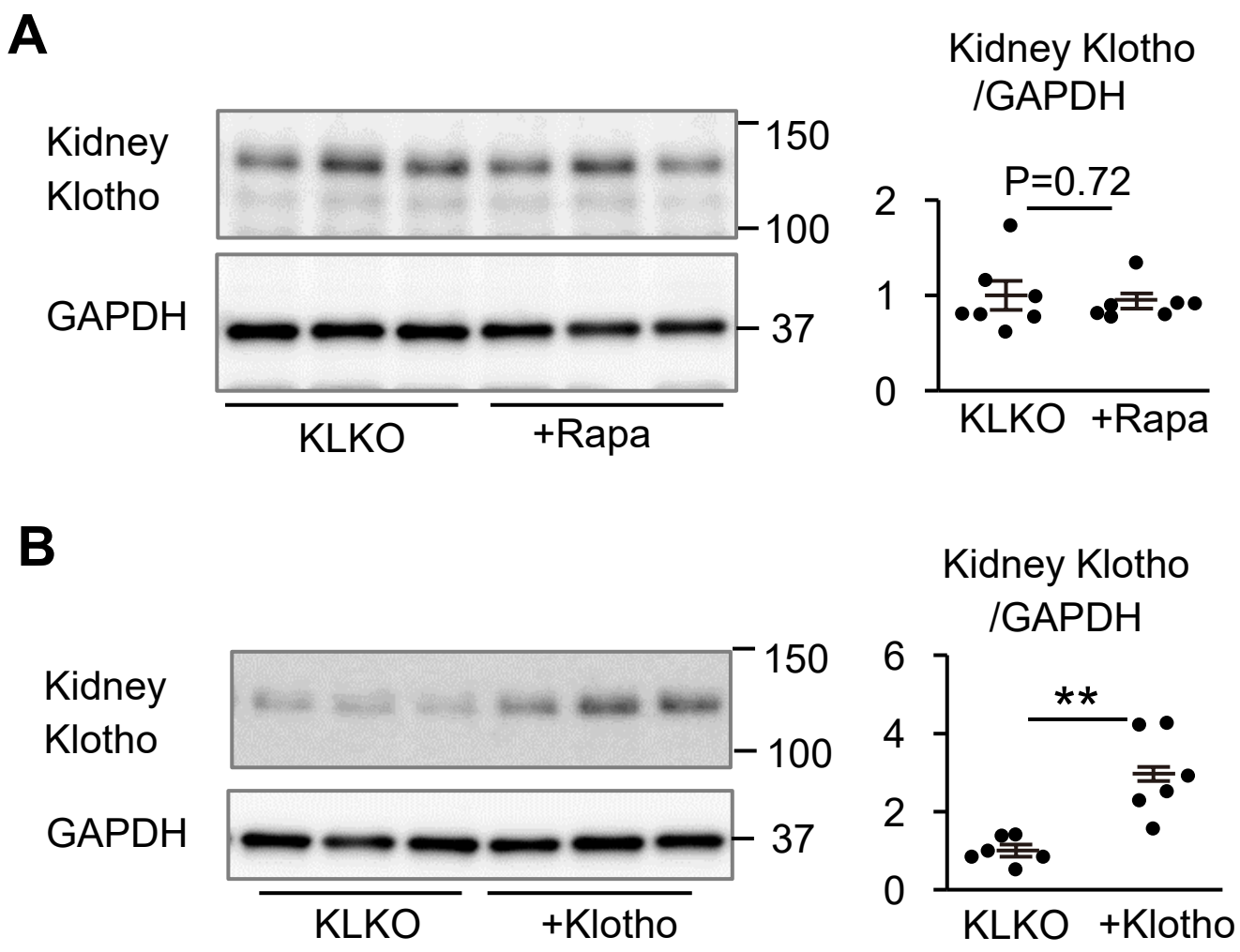

Supplementary Figure 3. Renal Klotho protein expression is not altered by rapamycin treatment but is restored by Klotho supplementation in KLhet mice.
(A) Representative Western blot and quantification of renal Klotho protein expression in whole-kidney lysates from KLhet mice and KLhet mice treated with rapamycin (+Rapa). GAPDH was used as a loading control. No significant difference was observed between groups (*n* = 7 per group; P = 0.72).
(B) Representative Western blot and quantification of renal Klotho protein expression in whole-kidney lysates from KLhet mice and KLhet mice treated with Klotho supplementation (+Klotho). Klotho expression was significantly increased following supplementation (*n* = 6 per group; **P < 0.01). Data are presented as mean ± SEM. Welch's t test was used for comparisons between two groups. **P < 0.01. N.S.

Supplementary Figure 4

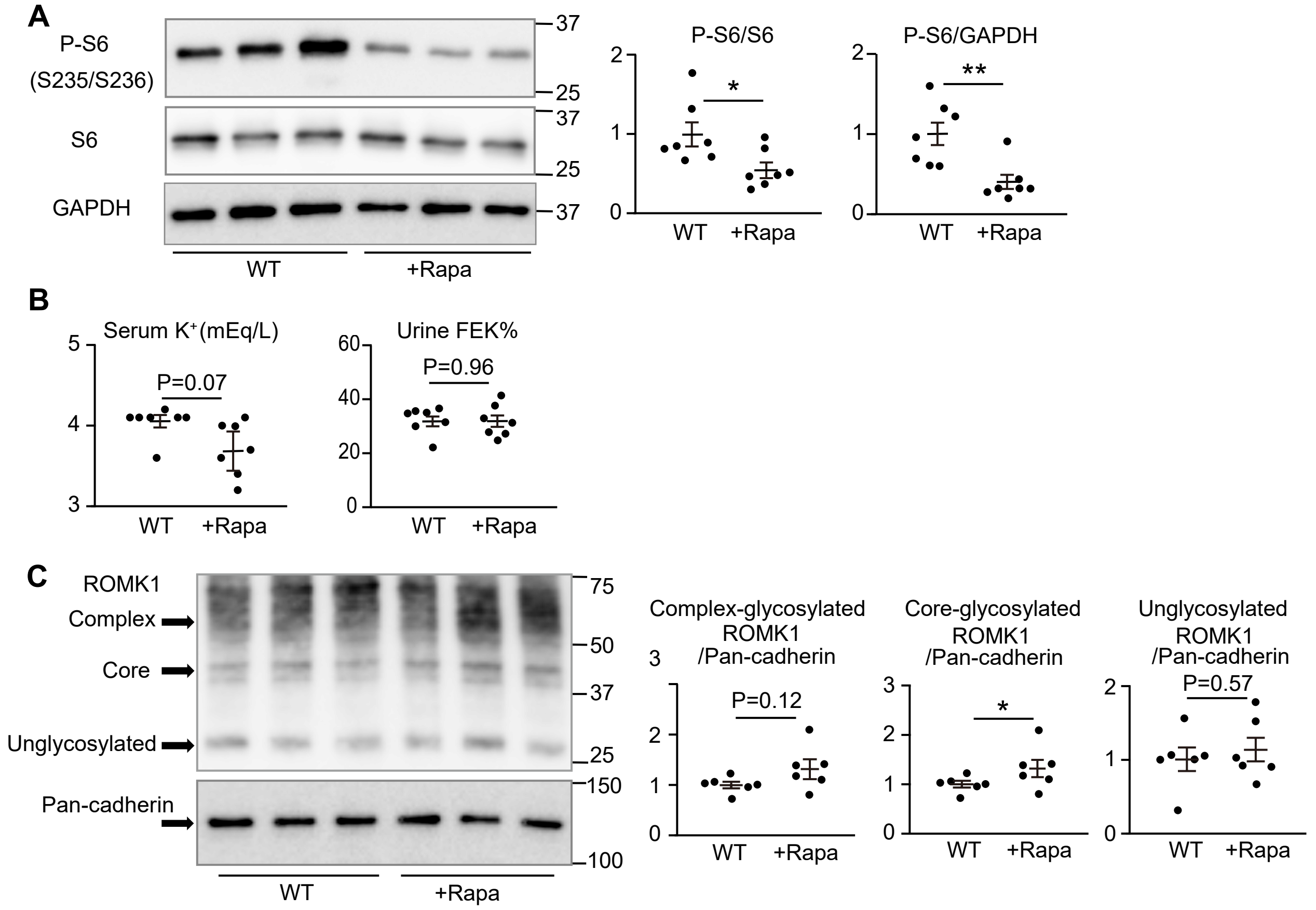

Supplementary Figure 4. Effects of rapamycin on renal mTORC1/S6 signaling, potassium handling, and ROMK1 expression in WT mice.

(A) Representative Western blot and quantification of phosphorylated S6 (pS6) expression in whole-kidney lysates from WT and rapamycin-treated WT (WT + Rapa) mice. pS6 levels were normalized to total S6 and GAPDH (n = 7 per group) .

(B) Fractional urinary potassium excretion (FEK) and serum potassium (K^+) concentrations in WT and WT + Rapa mice (n = 7 per group).

(C) Representative Western blot and quantification of ROMK1 protein expression in renal plasma membrane fractions. Complex-glycosylated, core-glycosylated, and unglycosylated forms are shown (n = 6 per group). Data are presented as mean ± SEM. Welch's t test was used for comparisons between two groups.

*P < 0.05, **P < 0.01, as indicated. Rapa, rapamycin.

Supplementary Figure 5

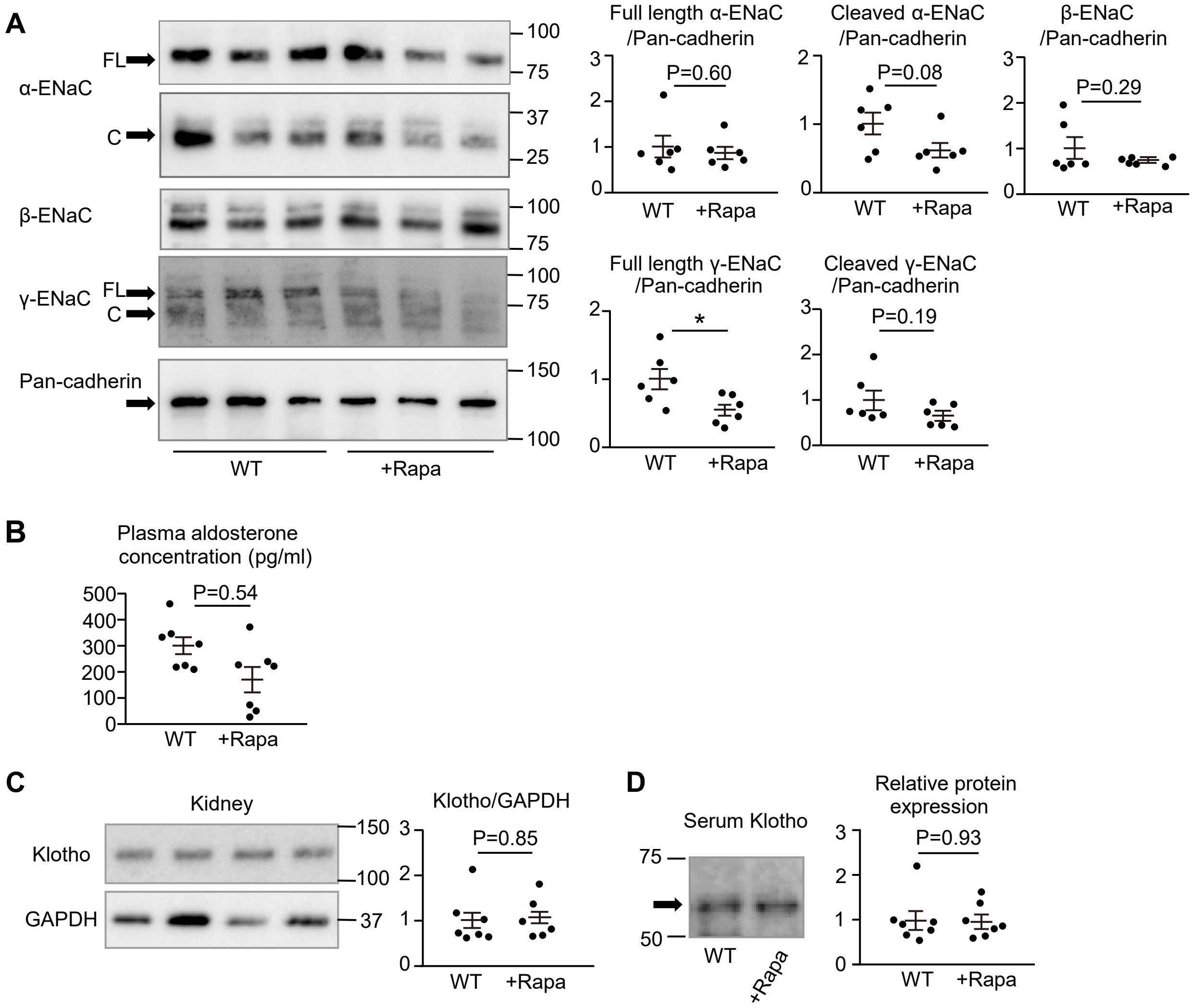

Supplementary Figure 5. Effects of rapamycin on ENaC expression, plasma aldosterone, and Klotho levels in WT mice.

(A) Representative Western blot and quantification of α-, β-, and γ-subunits of ENaC in renal plasma membrane fractions from WT and rapamycin-treated WT (WT + Rapa) mice. Full-length (FL) and cleaved (C) forms of α-ENaC and γ-ENaC, and full-length β-ENaC are shown (*n* = 6 per group) .

(B) Plasma aldosterone concentrations in WT and WT + Rapa mice (*n* = 7 per group).

(C) Representative Western blot and quantification of renal Klotho protein expression in whole-kidney lysates from WT and WT + Rapa mice (*n* = 7 per group).

(D) Serum Klotho concentrations in WT and WT + Rapa mice (*n* = 7 per group).

Data are presented as mean ± SEM. Welch's t test was used for comparisons between two groups. *P < 0.05, as indicated. FL, full-length; C, cleaved; Rapa, rapamycin.

Supplemental Figure 6

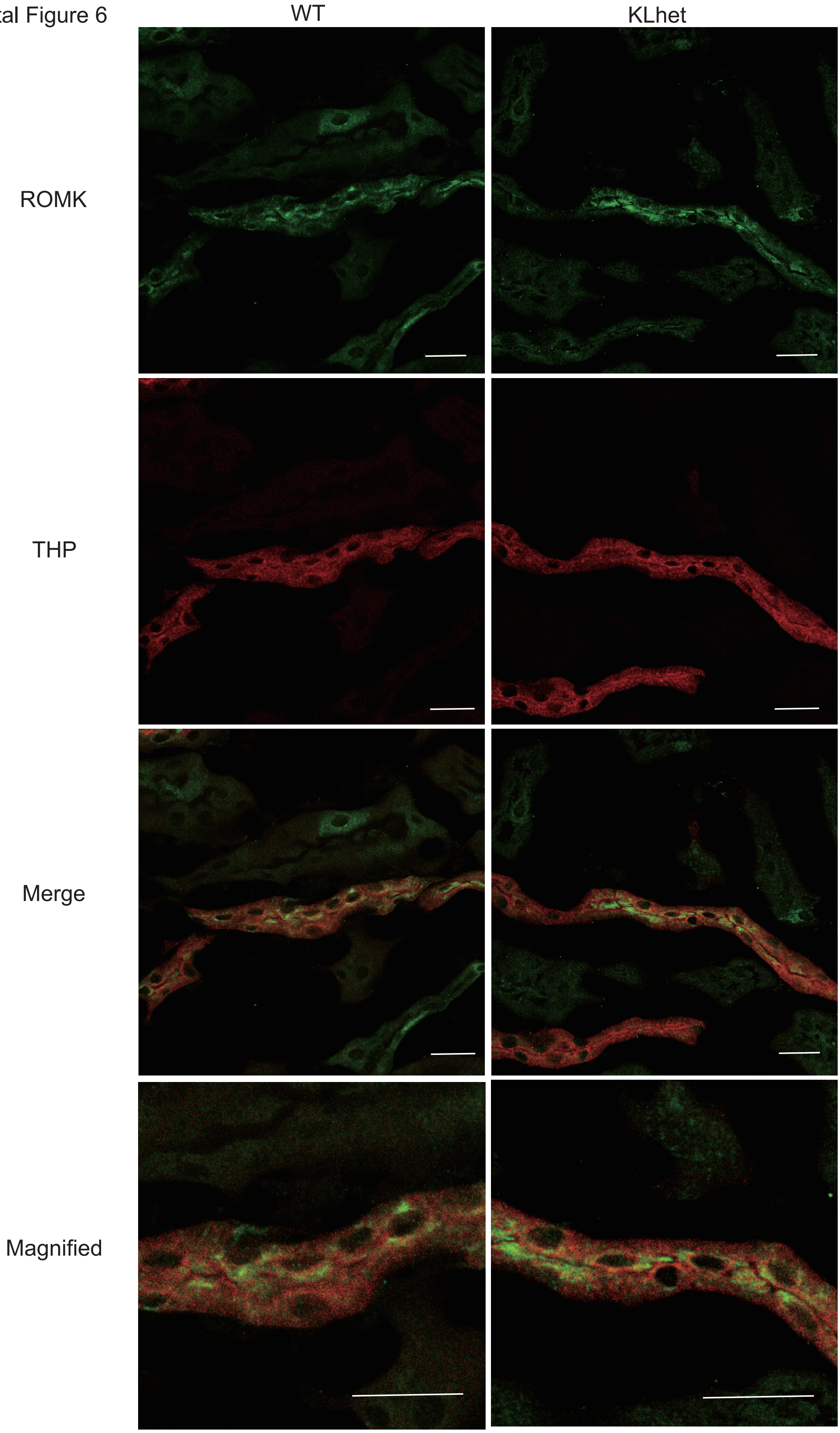

Supplementary Figure 6. ROMK expression in Tamm–Horsfall protein (THP)-positive thick ascending limb (TAL) segments is preserved in KLhet mice. Representative immunofluorescence staining for ROMK (green) and THP (red) in kidney sections from WT and KLhet mice. Merged images are shown in the third row. ROMK expression in THP-positive TAL segments was comparable between WT and KLhet mice, with no apparent reduction in KLhet mice. Magnified images (bottom row) show higher-magnification views of the indicated regions. Scale bars, 20 μm.

Supplementary Figure 7

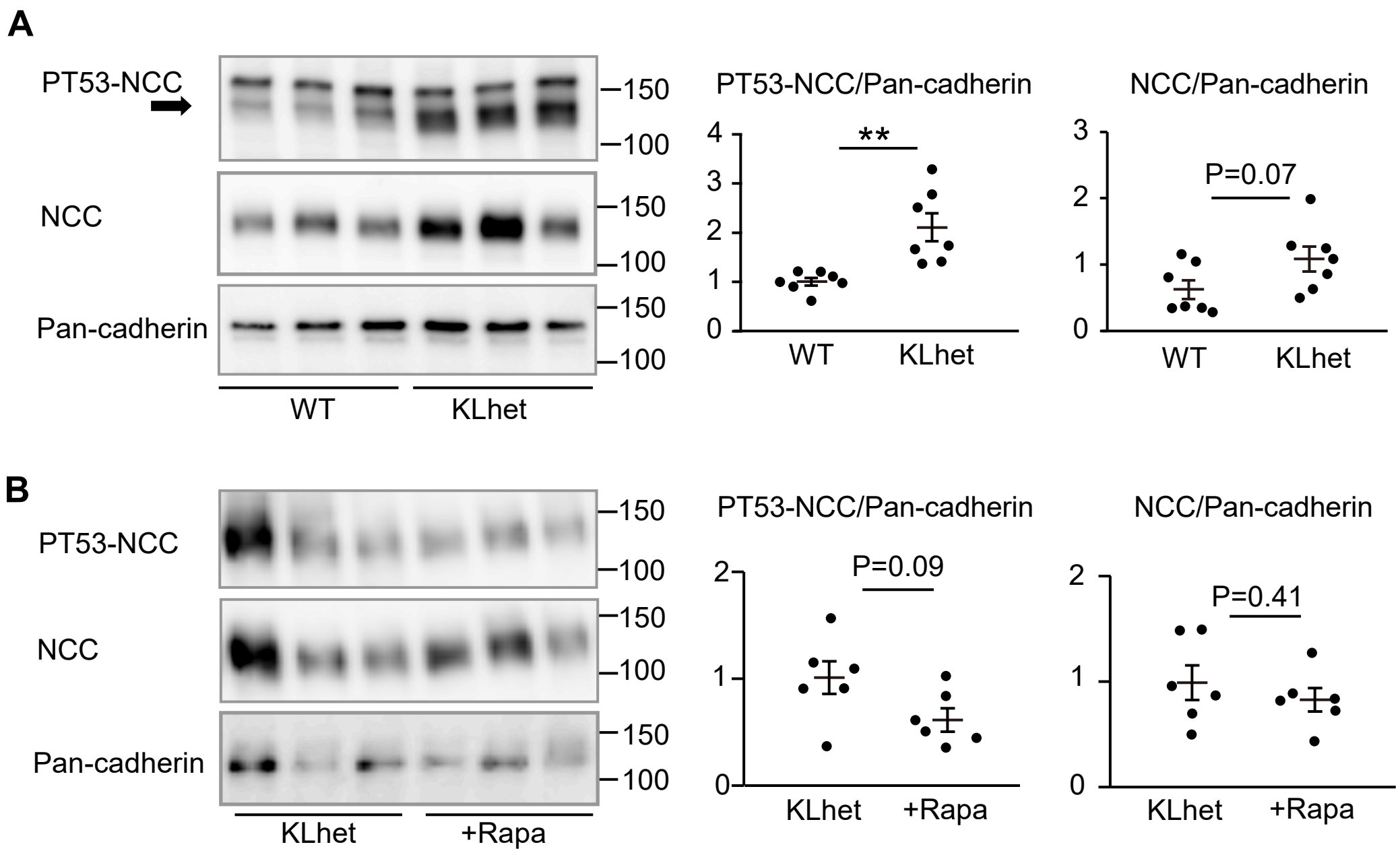

Supplementary Figure 7. NCC expression and phosphorylation in KLhet mice and the effects of rapamycin.

(A) Representative Western blot and quantification of phosphorylated NCC at Thr53 (pT53-NCC) and total NCC in renal plasma membrane fractions from WT and KLhet mice. pT53-NCC and total NCC were normalized to pan-cadherin (*n* = 7 per group).

(B) Representative Western blot and quantification of pT53-NCC and total NCC in renal plasma membrane fractions from KLhet and rapamycin-treated KLhet (KLhet + Rapa) mice. pT53-NCC and total NCC were normalized to pan-cadherin (*n* = 6 per group).

Supplementary Figure 8

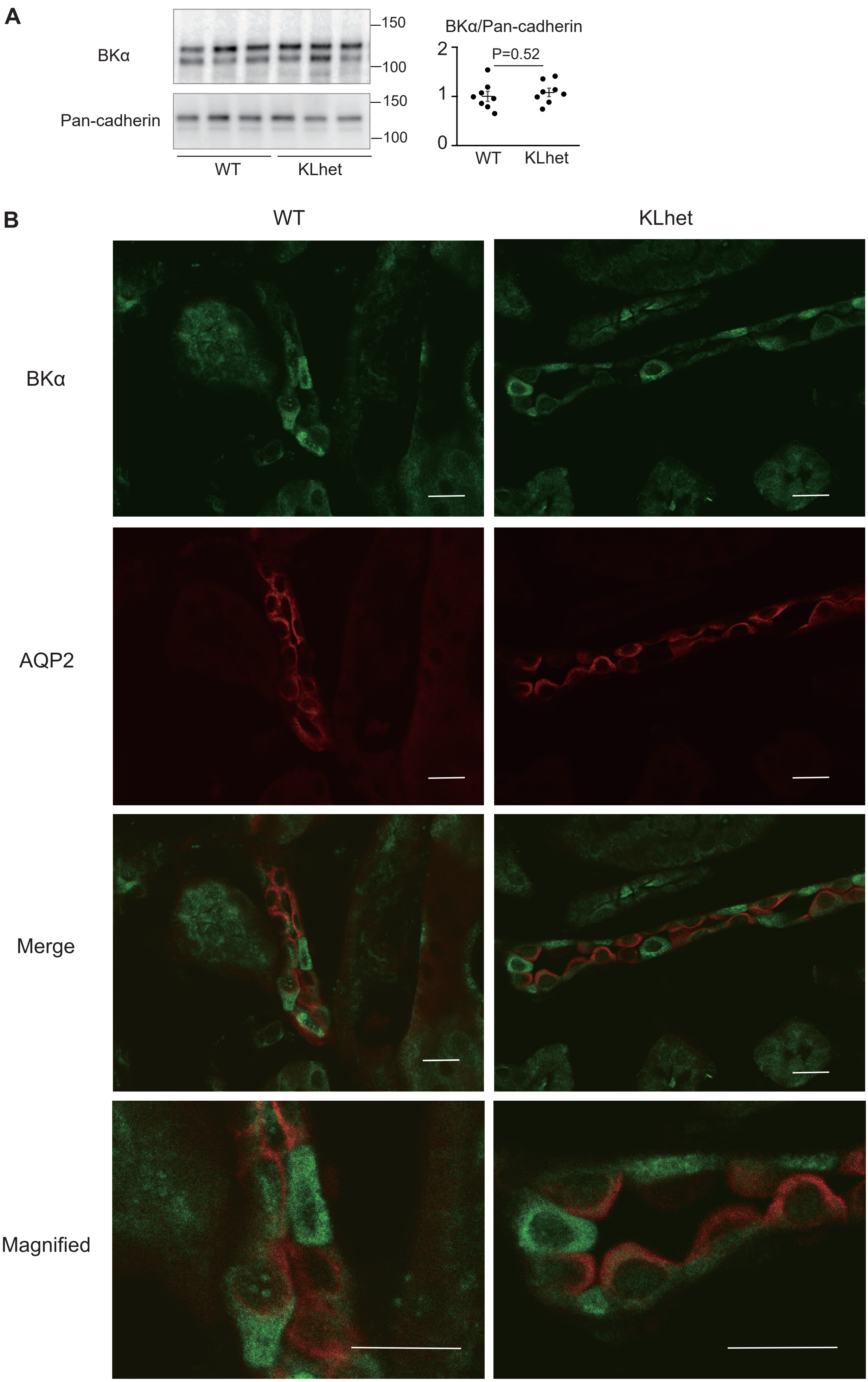

Supplementary Figure 8. Renal BKα expression in WT and KLhet mice.

(A) Representative Western blot and quantification of BKα protein expression in renal plasma membrane fractions from WT and KLhet mice. BKα expression was normalized to pan-cadherin (n = 8 per group).

(B) Double immunofluorescence staining for BKα (green) and aquaporin-2 (AQP2; red) in kidneys from WT and KLhet mice. Representative images and magnified views are shown. Scale bars, 20 μm.

Data are presented as mean ± SEM. Welch's t test was used for comparisons between two groups.
